# Arthroscopy versus mini-open surgery for femoroacetabular impingement: A systematic review of clinical outcomes

**DOI:** 10.1007/s00132-026-04779-z

**Published:** 2026-02-24

**Authors:** Filippo Migliorini, Nicola Maffulli, Manuel Giovanni Mazzoleni, Tommaso Bardazzi, Swaminathan Ramasubramanian, Naveen Jeyaraman, Madhan Jeyaraman

**Affiliations:** 1 Department of Trauma and Reconstructive Surgery, University Hospital of Halle, Martin-Luther University Halle-Wittenberg, Ernst-Grube-Street 40, 06097 Halle (Saale), Germany; 2 Department of Orthopaedic and Trauma Surgery, Academic Hospital of Bolzano (SABES-ASDAA), 39100 Bolzano, Italy; 3 Department of Life Sciences, Health, and Health Professions, Link Campus University of Rome, 00165 Rome, Italy; 4 Department of Trauma and Orthopaedic Surgery, Faculty of Medicine and Psychology, University La Sapienza, 00185 Roma, Italy; 5 School of Pharmacy and Bioengineering, Keele University Faculty of Medicine, ST4 7QB Stoke on Trent, UK; 6 Centre for Sports and Exercise Medicine, Barts and the London School of Medicine and Dentistry, Mile End Hospital, Queen Mary University of London, E1 4DG London, UK; 7 Department of Internal Medicine, Government Medical College, Omandurar Government Estate, 600018 Chennai, Tamil Nadu India; 8 Department of Orthopaedics, ACS Medical College and Hospital, Dr MGR Educational and Research Institute, 600077 Chennai, Tamil Nadu India

**Keywords:** Hip preservation, Cam morphology, Pincer morphology, Patient-reported outcomes, Reoperation rate, Hip pain, Functional outcome, Hüftgelenkerhalt, Cam-Morphologie, Pincer-Morphologie, Patient-Reported Outcomes, Reoperationsrate, Hüftschmerzen, Funktionelles Ergebnis

## Abstract

**Introduction:**

Arthroscopic and mini-open surgery are both valid options for managing symptomatic femoroacetabular impingement (FAI). The current systematic review compared the clinical outcomes of arthroscopic surgery versus mini-open surgery for femoroacetabular impingement. The primary outcomes assessed included patient-reported outcome measures (PROMs) and the incidence of complications.

**Material and methods:**

This systematic review followed the PRISMA statement. The Web of Science, PubMed, and Embase were accessed in June 2025 without additional filters or temporal constraints. All clinical studies concerning the surgical management of FAI were considered. Only investigations on arthroscopy or a mini-open surgical approach and those directly comparing the two techniques were included. Only studies with at least 6 months of follow-up were considered. The PROMs of interest were the visual analogue scale (VAS) and the modified Harris Hip Score (mHHS). Data on the reoperation rate and progression to total hip arthroplasty were collected. The mean difference (MD) and odds ratio (OR) measures were used for the comparisons.

**Results:**

Data from 233 clinical studies (54,272 patients) were retrieved. The mean follow-up duration was 33.9 ± 22.7 months. The mean age was 35.0 ± 5.2 years and the mean body mass index (BMI) was 25.1 ± 2.1 kg/m^2^. Arthroscopic surgery showed higher VAS (MD 1.2/10; *P* = 0.02) and mHHS values (MD 3.3/100; *P* < 0.01); however, despite being statistically significant, these differences had minimal clinical relevance. A statistically significant increase in the risk of reoperation was observed in the arthroscopy group (OR 8.9; *P* < 0.01). No significant difference was observed in the progression to THA (OR: 0.9; *P* = 0.4).

**Conclusion:**

Mini-open and arthroscopic surgery for symptomatic FAI achieve similar clinical outcomes and comparable complication rates at approximately 34 months follow-up. The mini-open approach was associated with a lower risk of reoperation. Future research should focus on long-term follow-up and standardized outcome measures to refine the understanding of these surgical options and enhance patient care.

**Supplementary Information:**

The online version of this article (https://doi.org/10.1007/s00132-026-04779-z) contains supplementary material, which is available to authorized users.

## Abstract (de)

**Einleitung:**

Sowohl arthroskopische als auch minimal-invasive Operationen sind geeignete Optionen zur Behandlung eines symptomatischen femoroazetabulären Impingements (FAI). Die vorliegende systematische Übersicht vergleicht die klinischen Ergebnisse arthroskopischer mit denen minimal-invasiver FAI-Operationen. Zu den primären Endpunkten gehörten Patient-Reported Outcome Measures (PROMs) und die Häufigkeit von Komplikationen.

**Material und Methoden:**

Diese systematische Übersicht erfolgte gemäß der PRISMA-Erklärung. Im Juni 2025 wurden die Datenbanken Web of Science, PubMed und Embase ohne zusätzliche Filter oder zeitliche Einschränkungen durchsucht. Alle klinischen Studien zur chirurgischen Behandlung von FAI wurden berücksichtigt. Es wurden nur Untersuchungen zu arthroskopischen oder mini-offenen chirurgischen Verfahren einbezogen sowie solche, die beide Techniken direkt miteinander verglichen. Es wurden nur Studien mit einer Nachbeobachtungszeit von mindestens 6 Monaten berücksichtigt. Die relevanten PROMs waren die visuelle Analogskala (VAS) und der modifizierte Harris Hip Score (mHHS). Es wurden Daten zur Reoperationsrate und zum Fortschreiten bis zur Hüfttotalendoprothese erhoben. Für die Vergleiche wurden die mittlere Differenz (MD) und die Odds-Ratio (OR) verwendet.

**Ergebnisse:**

Daten aus 233 klinischen Studien (54.272 Patienten) wurden ausgewertet. Die durchschnittliche Nachbeobachtungsdauer betrug 33,9 ± 22,7 Monate. Das Durchschnittsalter lag bei 35,0 ± 5,2 Jahren und der durchschnittliche Body-Mass-Index (BMI) bei 25,1 ± 2,1 kg/m^2^. Die arthroskopische Operation zeigte höhere VAS-Werte (MD 1,2/10; *p* = 0,02) und mHHS-Werte (MD 3,3/100; *p* < 0,01); diese Unterschiede waren jedoch trotz ihrer statistischen Signifikanz von minimaler klinischer Relevanz. In der Arthroskopiegruppe wurde ein statistisch signifikanter Anstieg des Risikos einer erneuten Operation beobachtet (OR 8,9; *p* < 0,01). Es wurde kein signifikanter Unterschied hinsichtlich der Progression zur Hüft-TEP beobachtet (OR: 0,9; *p* = 0,4).

**Schlussfolgerung:**

Mini-offene und arthroskopische Operationen bei symptomatischem FAI erzielen nach einer Nachbeobachtungszeit von etwa 34 Monaten ähnliche klinische Ergebnisse und vergleichbare Komplikationsraten. Der mini-offene Ansatz war mit einem geringeren Risiko einer erneuten Operation verbunden. Zukünftige Forschungsarbeiten sollten sich auf die Langzeitnachbeobachtung und standardisierte Messinstrumente konzentrieren, um die Kenntnisse dieser chirurgischen Optionen zu verfeinern und die Patientenversorgung zu verbessern.

## Introduction

Femoroacetabular impingement (FAI) is an abnormal contact between the femoral head and the acetabular rim, damaging the labrum and articular cartilage [1–3]. It is predominantly observed in young, active individuals and can lead to early-onset osteoarthritis if not appropriately treated [4–6]. Selecting an effective surgical strategy is crucial not only to prevent long-term joint deterioration but also to facilitate a timely and safe return to sport [7–9]. The primary types of FAI are cam, pincer and mixed impingement, each with distinct morphological abnormalities [10, 11]. Cam impingement arises from the aspherical femoral head, whereas pincer impingement results from acetabular overcoverage [12, 13]. Mixed impingement combines features of both cam and pincer types [14, 15]. The management of FAI has evolved over the past two decades, with surgical intervention becoming the mainstay treatment for patients who fail to respond to conservative management [16–18]. The original surgical approach involved hip dislocation for exposure of the femoral head and acetabulum to allow a complete correction of the deformity [19–21]. As cam impingement is usually in the anterolateral aspect of the hip joint [22, 23], in the modern era of hip joint preservation surgery, the most prevalent surgical approaches are arthroscopic and mini-open techniques [24, 25]. Arthroscopic surgery is a minimally invasive technique offering the benefits of reduced postoperative pain, shorter hospital stays and faster recovery times [26–28]. Conversely, mini-open surgery, performed through a minimally invasive anterior or anterolateral approach, typically combined with arthroscopic assistance, provides direct visualization and access to the hip joint, with more precise correction of joint deformities and soft tissue repair [29–31].

Despite the advances in surgical techniques and the growing body of literature, there remains considerable debate regarding the optimal surgical approach for FAI. The choice between arthroscopic and mini-open surgery often depends on various factors, including the surgeon’s expertise, patient-specific anatomical considerations and the extent of hip joint damage. Although arthroscopic surgery is less invasive and associated with quicker recovery, concerns have been raised about its efficacy in addressing bony deformities and preventing the progression of osteoarthritis. Conversely, mini-open surgery, while more invasive, might offer better correction and comprehensive treatment of associated pathologies, yet it is associated with higher morbidity and longer rehabilitation. Furthermore, there is a lack of consensus on standardized outcome measures, making comparison of results across studies challenging. Most studies use a variety of patient-reported outcome measures (PROMs), such as the modified Harris Hip Score (mHHS) and the Hip Outcome Score (HOS). Still, the heterogeneity in these measures complicates the synthesis of data sources. Additionally, long-term studies are needed to evaluate the sustainability of surgical outcomes and the risk of revision surgery or progression to total hip arthroplasty (THA).

The current systematic review compared clinical outcomes between arthroscopic surgery and mini-open surgery for FAI. The primary outcomes assessed included PROMs and the incidence of complications.

## Methods

### Eligibility criteria

All clinical studies concerning the surgical management of FAI were considered. Only investigations on arthroscopy or a mini-open surgical approach, and those directly comparing the two techniques, were included. Eligible studies must be published in peer-reviewed journals in one of the following languages: English, Italian, German, Spanish, or French. Only studies classified as levels I–IV of evidence were included, according to the 2020 Oxford Centre of Evidence-Based Medicine [32]. Opinions, editorials, letters, and reviews were excluded. Moreover, studies involving animals, in vitro experiments, computational analyses, biomechanical assessments, or cadaveric research were disregarded. Only studies with a minimum of 6 months of follow-up were eligible for inclusion. Studies which compared a failure group and a success group were excluded.

### Search strategy

The present systematic review followed the guidelines outlined in the 2020 Preferred Reporting Items for Systematic Reviews and Meta-Analyses (PRISMA) statement [33]. The literature search followed the PICOTD algorithm:

- P (Problem): FAI;
- I (Intervention): surgical management;
- C (Comparison): arthroscopy and mini-open;
- O (Outcomes): PROMs and complications;
- T (Timings): minimum 6 months of follow-up;
- D (Design): clinical study.

PubMed, Web of Science, and Embase were accessed in June 2025 without additional filters or temporal constraints. The Medical Subject Headings (MeSH) used in the database search are reported in the Appendix.

### Selection and data collection

The database search was conducted by two authors (MGM and TB). All retrieved titles were manually screened and the abstracts were reviewed if deemed relevant. Full texts were scrutinized when judged relevant. Articles lacking accessible full texts were excluded. Furthermore, a cross-reference of the bibliographies of full-text articles was conducted to identify potential candidates for inclusion. Any discrepancies were resolved by a third senior author (**), who made the final decision.

### Data items

Data extraction was conducted by two authors (MGM and TB). The following general information was collected for each study: author, year of publication, journal, and length of follow-up. The following baseline data were extracted: the number of patients, the number of women and BMI. Data concerning the visual analogue scale (VAS) and modified Harris Hip Score (mHHS) [34] were collected at baseline and at the last follow-up. The minimally clinically important differences (MCID) for the VAS and mHHS were 1.5 and 14.6, respectively [35–38]. Data concerning the following complications were retrieved: reoperations and progression to THA. Extraction was performed using Microsoft Office Excel version 16.0 (Microsoft Corporation, Redmond, USA).

### Assessment of the risk of bias

The risk of bias assessment followed the guidelines outlined in the Cochrane Handbook for Systematic Reviews of Interventions [39]. Two authors (MJ and NJ) evaluated the risk of bias in the extracted studies. Nonrandomized controlled trials (non-RCTs) were evaluated using the Risk of Bias in Nonrandomised Studies of Interventions (ROBINS-I) tool [40]. The tool considers seven domains of potential bias. These domains include confounding factors and patient selection characteristics before the comparative intervention, bias in classification during the intervention, methodological quality of the post-intervention comparison, which involves deviations from the intended interventions, missing data, inaccurate outcome measurement and bias in the selection of reported outcomes. The ROBINS‑I chart was generated using the RobVis software (Risk-of-bias VISualization; RiskofBias.info, Bristol, UK) [41]. Randomized controlled trials (RCTs) were assessed using the revised Risk of Bias (RoB2) [42, 43] of the Cochrane Risk of Bias in Randomised Trials (RoB) [44]. The following endpoints were considered: bias arising from the randomization process, bias from deviations from intended interventions, bias due to missing outcome data, bias in the measurement of the outcome, and bias in the selection of the reported result.

### Synthesis method

The main author (FM) performed the statistical analyses following the recommendations of the Cochrane Handbook for Systematic Reviews of Interventions [39]. The IBM SPSS Statistics version 25 (International Business Machines Corporation, Armonk, NY, USA) was used to perform descriptive statistics. The arithmetic mean and standard deviation were used for continuous data, and the frequency (events/observations) for dichotomous variables.

## Results

### Study selection

**Fig. 1:**
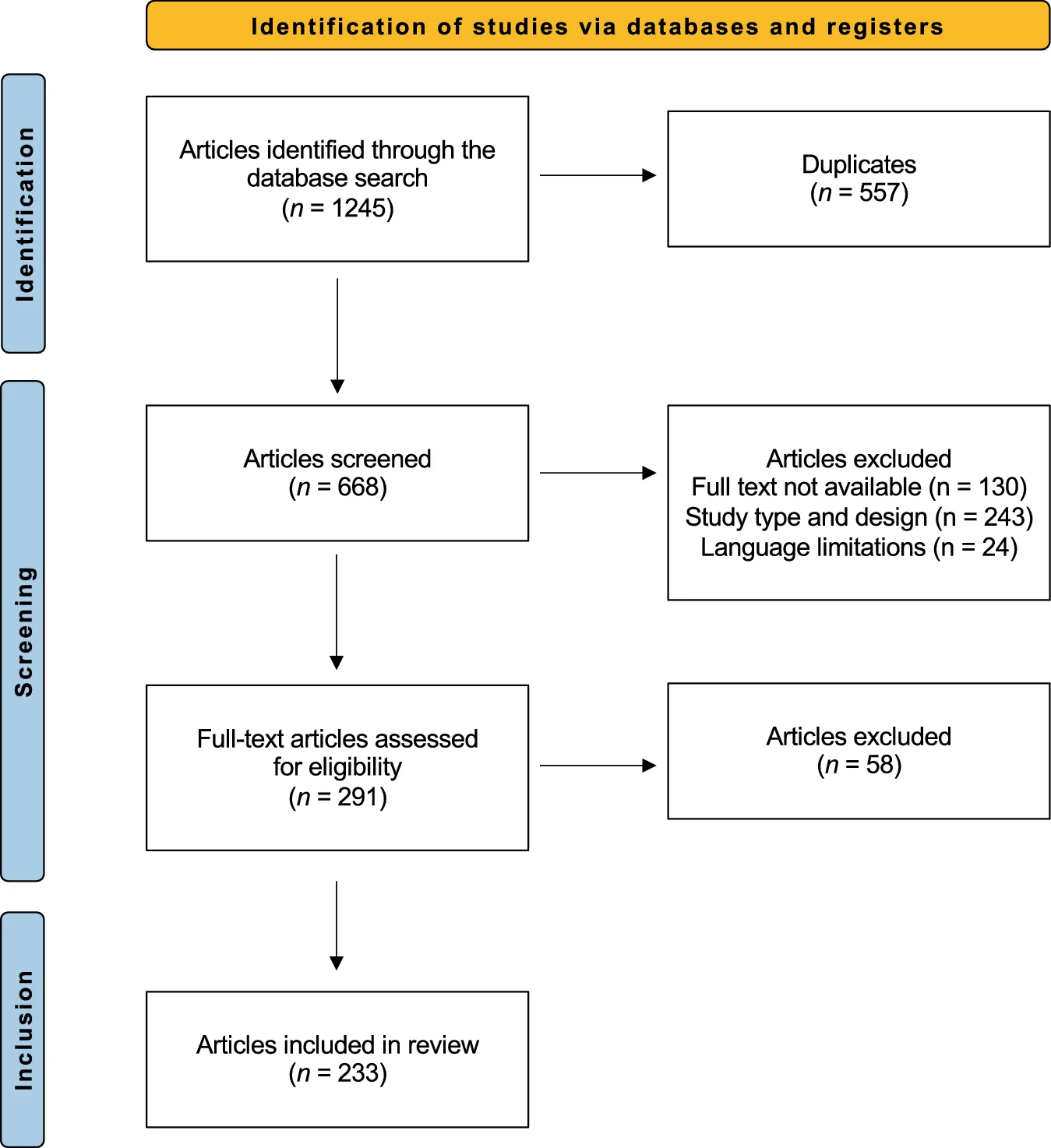
PRISMA flow chart of the literature search

The systematic literature search identified 1245 articles. After removing duplicates, we screened the abstracts of 688 articles for eligibility. A total of 397 articles were excluded for various reasons. These included a mismatch with the predefined study design criteria (*N* = 243), no full-text available (*N* = 130), and language barriers (*N* = 24). A further 58 articles were excluded after a full-text review of the remaining 291. Consequently, this systematic review included a final selection of 233 studies. The results of the literature search are shown in Fig. 1.

### Risk of bias assessment

**Fig. 2:**
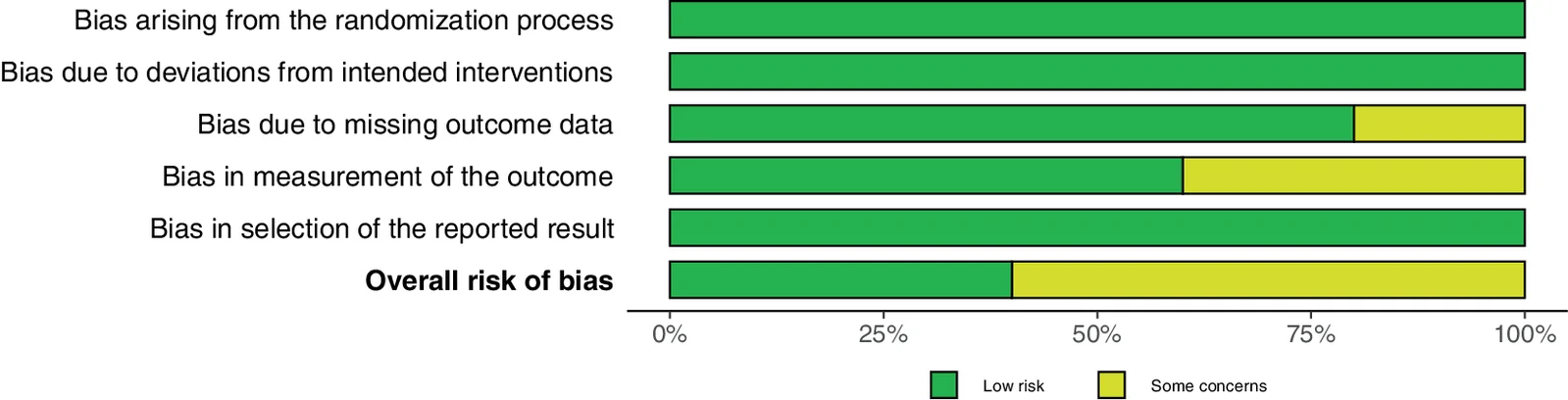
The RoB2 of RCTs

Of the studies included in this systematic review five qualified as RCTs. The Cochrane risk-of-bias assessment tool (ROB 2) was used to evaluate these five RCTs. The analysis revealed good baseline comparability between the intervention and control groups across all studies, indicating a low risk of bias introduced by randomization. Furthermore, the assessment identified no significant concerns regarding deviations from the intended intervention protocol or selective outcome reporting; however, two of the five RCTs exhibited a moderate risk of bias in outcome measurement because of a lack of blinding of assessors. Additionally, one trial was rated at moderate risk of bias due to missing data. Consequently, three of the five RCTs were judged to have a moderate overall risk of bias, whereas the remaining two demonstrated a low risk of bias (Fig. 2).

**Fig. 3:**
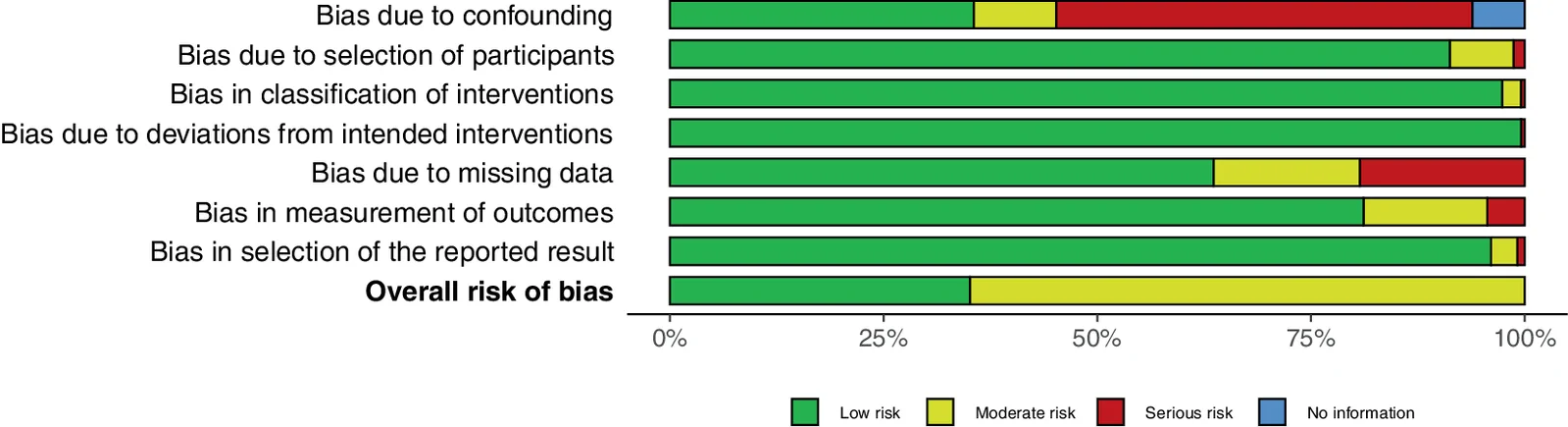
The ROBINS‑I of non-RCTs

The ROBINS‑I tool was used to assess the risk of bias in the non-RCTs. A critical finding emerged in the first domain, with a substantial proportion (two thirds) of studies exhibiting a serious or moderate risk of bias due to confounding. This highlights a key limitation in the methodological quality of these studies. Encouragingly, the risk of bias arising from participant selection remained low across most studies. Furthermore, a low risk of bias was maintained in nearly all studies with respect to both the classification of interventions and adherence to the intended intervention protocol; however, some concerns regarding missing data were identified within the domains assessing postintervention bias. The selection of reported results presented minimal concerns in almost all studies. In conclusion, the ROBINS‑I assessment indicated a moderate or low overall risk of bias across the non-RCT studies, suggesting an acceptable level of methodological quality, although the potential confounding bias (Fig. 3).

### Study characteristics and results of individual studies

Of the 233 included studies, 221 utilized an arthroscopic technique, 10 utilized a mini-open surgical approach and 2 directly compared the 2 methodologies. Data on 54,272 patients were retrieved; 51,948 underwent arthroscopy and 2324 underwent surgery via a mini-open approach. The mean follow-up duration was 33.9 ± 22.7 months. The mean age was 35.0 ± 5.2 years, and the mean BMI was 25.1 ± 2.1 kg/m^2^. The general characteristics of the included studies are presented in the appendix.

**Table 1:** Baseline comparability

Endpoint	Arthroscopy (*N* = 51,948)	Mini-open (*N* = 2324)	*P*
Mean follow-up (*months*)	32.5 ± 22.0	56.5 ± 22.1	0.05
Mean age	35.0 ± 5.2	34.1 ± 5.0	0.3
Mean BMI	25.0 ± 2.1	26.4 ± 1.3	0.07
Female (*%*)	54.9% (28,509 of 48,028)	48.0% (934 of 1944)	0.05
mHHS (*mean*)	62.6 ± 15.3	57.2 ± 8.9	0.07

*mHHS* modified Harris Hip Score,* BMI* Body mass index

### Baseline comparability

Between the two groups, baseline comparability was evidenced in the mean length of follow-up, mean age, mean BMI, female/male ratio, and mHHS (Table 1).

**Table 2:** Results of PROMs

Endpoint	Arthroscopic (*N* = 51948)	Mini-open (*N* = 2324)	Mean difference	95% CI	*P*
VAS (*mean*)	2.7 ± 3.3	1.5 ± 2.2	1.2	0.16–2.24	0.02
mHHS (*mean*)	84.8 ± 16.8	81.5 ± 13.2	3.3	2.47–4.13	< 0.0001

*mHHS* modified Harris Hip Score; *VAS* visual analogue scale; *CI* confidence interval

### Synthesis of results

Arthroscopic surgery showed significantly higher VAS (MD 1.2; 95% CI 0.16–2.24; *P* = 0.02) and higher mHHS (MD 3.3; 95% CI: 2.47; 4.13; *P* < 0.01; Table 2).

**Table 3:** Results of complications

Endpoint	Arthroscopic % (*N* = 51,948)	Mini-open % (*N* = 2324)	Odds ratio	95% CI	*P*
Reoperation	6.5 (856 of 13200)	0.8 (10 of 1289)	8.87	4.74 to 16.59	< 0.0001
Progression to THA	6.9 (817 of 11838)	7.5 (132 of 1765)	0.92	0.76 to 1.11	0.4

*THA* total hip arthroplasty

A significantly increased risk of reoperation was observed in the arthroscopy group (OR 8.87; 95% CI 4.47 to 16.59; *P* < 0.0001). No significant difference was observed in the progression to THA (OR 0.92; 95% CI 0.76 to 1.11; *P* = 0.4; Table 3).

## Discussion

The present systematic review aimed to compare clinical outcomes between arthroscopic surgery and mini-open surgery for FAI, focusing on PROMs and complication incidence. The main findings indicate a statistically significant difference in PROMs between the two surgical techniques at approximately 34 months of follow-up; however, as these differences did not overcome the MCID [35–38] the clinical relevance is marginal. The arthroscopy group had a significantly higher risk of reoperation, whereas conversion to THA rates were comparable.

The primary outcome measures in the present systematic review included the VAS and the modified mHHS. The analysis revealed marginally better results for the mHHS and a slight disadvantage for the VAS. Arthroscopic surgery appears to yield greater improvements in hip function at the expense of less pain relief in the medium term; however, the minor mean differences, especially for the mHHS and the apparent contradiction between the two results do not enable the establishment of a clear clinical relevance. Finally, despite mHHS being one of the most commonly used outcome measures worldwide, its value in assessing young, active patients with hip disorders has recently been questioned as it was initially designed for hip osteoarthritic conditions [45, 46]. Arthroscopic surgery has been lauded for its minimally invasive nature, which typically results in reduced postoperative pain, shorter hospital stays, and faster recovery times compared to more invasive techniques; however, concern has been raised that arthroscopic surgery may not fully address the bony deformities associated with FAI and could be less effective than a mini-open approach in preventing the progression of osteoarthritis [47]. This is partly supported by the higher VAS score observed in the present results; however, the greater functional improvement observed appears to mitigate these concerns, suggesting that arthroscopy can yield optimal results. While more invasive, mini-open surgery enables direct visualization and access to the hip joint, potentially enabling more precise correction of bony abnormalities [29, 48]. This approach might be expected to yield superior long-term outcomes because of easier exposure to the pathological features of FAI; however, during the approximately 34-month follow-up period, mini-open surgery did not confer additional PROM benefits compared with arthroscopic surgery, except for a slightly lower VAS. This apparent contradiction in outcomes underscores the importance of surgical expertise and individualized patient assessment in determining the most appropriate surgical approach.

The rate of reoperation was significantly lower for the mini-open approach, while progression to THA was comparable between the two surgical techniques. The lower incidence of reoperation with the mini-open approach can be explained by the previously mentioned possibility of more precise correction of bony abnormalities, owing to better visualization. Anyway, the rates were low for both techniques indicating that they are generally safe and effective in the medium term. The rate of progression to THA was comparable, suggesting that neither method significantly increases the risk of requiring further surgical intervention within the follow-up period. These findings align with previous studies reporting similar complication rates between the two surgical approaches [49–53]. The low complication rates observed in the present systematic review support the continued use of arthroscopic and mini-open surgeries as viable options for managing FAI; however, the potential for selection bias should be considered, as surgeons may preferentially select one technique over the other based on patient characteristics not fully accounted for in the included studies.

Several limitations must be acknowledged when interpreting the results of this systematic review. A quantitative meta-analysis could not be performed, as only a limited number of studies directly compared arthroscopic and mini-open surgery. In contrast, most included investigations reported outcomes for a single technique without a comparator. The synthesis was therefore based on aggregated descriptive outcomes, which should be interpreted as an overview of trends rather than as definitive comparative effect estimates. The heterogeneity of the included studies, particularly with respect to baseline characteristics, surgical techniques and outcome measures, poses a significant challenge for data synthesis. Moreover, only two of the included studies directly compared the mini-open approach and arthroscopic surgery. Finally, the mini-open cohort was much smaller, which may have underpowered the comparisons. Residual confounding factors may still influence the results despite efforts to ensure baseline comparability. Although adequate for assessing medium-term outcomes, the approximately 34-month follow-up period may not capture the long-term efficacy and sustainability of the surgical interventions. Long-term follow-up studies are necessary to determine whether the observed equivalence in outcomes persists over time and to identify any differences in the progression of osteoarthritis or in the need for revision surgery. The reliance on PROMs introduces subjectivity and variability in outcome assessment. While PROMs are valuable for capturing patient perspectives on pain and function, they are influenced by patient groups, individual expectations, cultural factors, and reporting biases. The included studies also reported few PROMs, which could have yielded more comparable results. For the mini-open cohort, no studies reported on PROMs, such as the International Hip Outcome Tool (iHOT), the Hip Outcome Score-Activity of Daily Living (HOS-ADL), or the sport-specific subscale (HOS-SSS). This prevented a more in-depth comparison and allowed comparison only of VAS and mHHS, whose values have recently been questioned, as already stated [45, 46]. Future studies should incorporate more appropriate PROMs suitable for young active patients, and objective measures, such as imaging and biomechanical assessments, to provide a more comprehensive evaluation of surgical outcomes.

The findings of this review have several implications for clinical practice. The better results in VAS and rates of reintervention demonstrated by the mini-open approach seem to suggest it could be better to address and correct bony deformities, but they could also be attributed to a steeper learning curve for arthroscopy. On the other hand, arthroscopy appears to yield better functional outcomes and is a valuable alternative. The surgical approach should be tailored to the individual patient, considering factors such as the severity and morphology of the impingement, the presence of comorbidities, associated soft tissue lesions, patient preferences and the surgeon’s expertise and learning curve. For patients and clinicians the results provide reassurance that both surgical options can yield satisfactory outcomes with respect to pain relief and functional improvement. The decision-making process should involve a thorough discussion of the potential risks and benefits of each approach, with consideration of the patient’s specific circumstances and goals.

Future research should focus on several key areas to build on the findings of this review. Long-term studies with extended follow-up periods are essential to assess the durability of surgical outcomes and the potential for late complications or the need for revision surgery. Additionally, standardizing appropriate outcome measures across studies would facilitate more accurate comparisons and meta-analyses, thereby enhancing the overall quality of evidence in this field. Research should also explore the role of emerging technologies and techniques in managing FAI. For example, the use of advanced imaging modalities and computer-assisted surgical planning could improve the precision of arthroscopic and mini-open surgeries. Comparative studies evaluating the effectiveness of these innovations could provide valuable insights into their potential benefits and limitations.

## Conclusion

Mini-open and arthroscopic surgery for symptomatic FAI achieve similar clinical outcomes and a comparable complication rate at approximately 34 months of follow-up. The mini-open approach was associated with a lower risk of reoperation. Future research should focus on long-term follow-up and standardized outcome measures to refine the understanding of these surgical options and enhance patient care.

### Consent to participate

Not applicable.

### Consent to publish

Not applicable.

### Use of AI

Language editing was performed using Grammarly (Superhuman Platform Inc., San Francisco, CA, USA).

### Registration and protocol

The present review was not registered.

## Supplementary Information

ESM1: Research question

ESM2: Appendix

## Data Availability

The datasets generated during and/or analyzed during the current study are available throughout the manuscript.

## Abbreviations

FAI: Femoroacetabular impingement
HOS: Hip Outcome Score
mHHS: modified Harris Hip Score
PROMs: Patient-reported outcome measures
THA: Total hip arthroplasty
