# Supplementary material for: Arthroscopy versus mini-open surgery for femoroacetabular impingement: A systematic review of clinical outcomes
Source: Orthopadie (Heidelb). 2026 Feb 24;55(9):737–44. doi: 10.1007/s00132-026-04779-z (PMC13524977; doi:10.1007/s00132-026-04779-z)
Supplement: Supplementary file 1 [file 132_2026_4779_MOESM1_ESM.pdf]

# Research Question:
**Femoracetabular Impingement Mini-Open vs Arhtroscopy**

## Concept 1: Condition

**Keywords:**
Acetabulum
cam
Cam impingement
cam lesion
cam type
FAI
FAI syndrome
FAIS
Femoracetabular impingement
Femoro-acetabular impingement
Hip joint
hip pain
Pincer
pincer hip
Pincer impingement
pincer type

**Mesh:**
"Acetabulum"[Mesh]
"Acetabulum / injuries"[Mesh]
"Acetabulum / pathology"[Mesh]
"Cartilage / physiopathology"[Mesh]
"Femoracetabular Impingement"[Mesh]
"Femoracetabular Impingement / diagnosis"[Mesh]
"Femoracetabular Impingement / etiology"[Mesh]
"Femoracetabular Impingement / pathology"[Mesh]
"Femoracetabular Impingement / physiopathology"[Mesh]
"Hip"[Mesh]
"Hip Joint / pathology"[Mesh]
"Joint Diseases / pathology"[Mesh]
"Pain"[Mesh]

## Concept 2: Therapy

**Keywords:**
acetabular labral refixation
acetabular labral segmental reconstruction
Arthroscopic assisted mini-open approach
arthroscopic labral reconstruction
arthroscopic surgery
arthroscopic surgical procedures
Arthroscopy
hip arthroscopic surgery
Hip arthroscopy
labral reconstruction
labral repair
Mini-open Hueter approach
Reconstruction

**Mesh:**
"Acetabulum / surgery"[Mesh]
"Arthroscopy"[Mesh]
"Arthroscopy / methods"[Mesh]
"Arthroscopy / standards"[Mesh]
"Cartilage / surgery"[Mesh]
"Debridement"[Mesh]
"Femoracetabular Impingement / surgery"[Mesh]
"Hip Joint / surgery"[Mesh]
"Joint Diseases / surgery"[Mesh]
"Minimally Invasive Surgical Procedures"[Mesh]

## Concept 3: Interest of the outcome PROMS

**Keywords:**
MCID
minimal clinically important difference (MCID)
patient outcomes
Patient Reported Outcome Measures
Treatment outcome
VAS
visual analog scale

**Mesh:**
"Minimal Clinically Important Difference"[Mesh]
"Pain Measurement / methods"[Mesh]
"Patient Outcome Assessment"[Mesh]

"Patient Reported Outcome Measures"[Mesh] "Visual Analog Scale"[Mesh]
"Patient Satisfaction"[Mesh]

## Searching Strategy

"Acetabulum"[Mesh] OR "Acetabulum / injuries"[Mesh] OR "Acetabulum / pathology"[Mesh] OR "Cartilage / physiopathology"[Mesh] OR "Femoracetabular Impingement"[Mesh] OR "Femoracetabular Impingement / diagnosis"[Mesh] OR "Femoracetabular Impingement / etiology"[Mesh] OR "Femoracetabular Impingement / pathology"[Mesh] OR "Femoracetabular Impingement / physiopathology"[Mesh] OR "Hip"[Mesh] OR "Hip Joint / pathology"[Mesh] OR "Joint Diseases / pathology"[Mesh] OR "Pain"[Mesh] OR Acetabulum OR cam OR Cam impingement OR cam lesion OR cam type OR FAI OR FAI syndrome OR FAIS OR Femoracetabular impingement OR Femoro-acetabular impingement OR Hip joint OR hip pain OR Pincer OR pincer hip OR Pincer impingement OR pincer type

**AND**

"Acetabulum / surgery"[Mesh] OR "Arthroscopy"[Mesh] OR "Arthroscopy / methods"[Mesh] OR "Arthroscopy / standards"[Mesh] OR "Cartilage / surgery"[Mesh] OR "Debridement"[Mesh] OR "Femoracetabular Impingement / surgery"[Mesh] OR "Hip Joint / surgery"[Mesh] OR "Joint Diseases / surgery"[Mesh] OR "Minimally Invasive Surgical Procedures"[Mesh] OR acetabular labral refixation OR acetabular labral segmental reconstruction OR Arthroscopic assisted mini-open approach OR arthroscopic labral reconstruction OR arthroscopic surgery OR arthroscopic surgical procedures OR Arthroscopy OR hip arthroscopic surgery OR Hip arthroscopy OR labral reconstruction OR labral repair OR Mini-open Hueter approach OR Reconstruction

**AND**

"Minimal Clinically Important Difference"[Mesh] OR "Pain Measurement / methods"[Mesh] OR "Patient Outcome Assessment"[Mesh] OR "Patient Reported Outcome Measures"[Mesh] OR "Patient Satisfaction"[Mesh] OR "Visual Analog Scale"[Mesh] OR MCID OR minimal clinically important difference (MCID) OR patient outcomes OR Patient Reported Outcome Measures OR Treatment outcome OR VAS OR visual analog scale

**SUMMARY**

("Acetabulum"[Mesh] OR "Acetabulum / injuries"[Mesh] OR "Acetabulum / pathology"[Mesh] OR "Cartilage / physiopathology"[Mesh] OR "Femoracetabular Impingement"[Mesh] OR "Femoracetabular Impingement / diagnosis"[Mesh] OR "Femoracetabular Impingement / etiology"[Mesh] OR "Femoracetabular Impingement / pathology"[Mesh] OR "Femoracetabular Impingement / physiopathology"[Mesh] OR "Hip"[Mesh] OR "Hip Joint / pathology"[Mesh] OR "Joint Diseases / pathology"[Mesh] OR "Pain"[Mesh] OR Acetabulum OR cam OR Cam impingement OR cam lesion OR cam type OR FAI OR FAI syndrome OR FAIS OR Femoracetabular impingement OR Femoro-acetabular impingement OR Hip joint OR hip pain OR Pincer OR pincer hip OR Pincer impingement OR pincer type) AND ("Acetabulum / surgery"[Mesh] OR "Arthroscopy"[Mesh] OR "Arthroscopy / methods"[Mesh] OR "Arthroscopy / standards"[Mesh] OR "Cartilage / surgery"[Mesh] OR "Debridement"[Mesh] OR "Femoracetabular Impingement /

surgery"[Mesh] OR "Hip Joint / surgery"[Mesh] OR "Joint Diseases / surgery"[Mesh] OR "Minimally Invasive Surgical Procedures"[Mesh] OR acetabular labral refixation OR acetabular labral segmental reconstruction OR Arthroscopic assisted mini-open approach OR arthroscopic labral reconstruction OR arthroscopic surgery OR arthroscopic surgical procedures OR Arthroscopy OR hip arthroscopic surgery OR Hip arthroscopy OR labral reconstruction OR labral repair OR Mini-open Hueter approach OR Reconstruction) AND ("Minimal Clinically Important Difference"[Mesh] OR "Pain Measurement / methods"[Mesh] OR "Patient Outcome Assessment"[Mesh] OR "Patient Reported Outcome Measures"[Mesh] OR "Patient Satisfaction"[Mesh] OR "Visual Analog Scale"[Mesh] OR MCID OR minimal clinically important difference (MCID) OR patient outcomes OR Patient Reported Outcome Measures OR Treatment outcome OR VAS OR visual analog scale)
