# Supplementary material for: Arthroscopy versus mini-open surgery for femoroacetabular impingement: A systematic review of clinical outcomes
Source: Orthopadie (Heidelb). 2026 Feb 24;55(9):737–44. doi: 10.1007/s00132-026-04779-z (PMC13524977; doi:10.1007/s00132-026-04779-z)
Supplement: Supplementary file 2 [file 132_2026_4779_MOESM2_ESM.pdf]

APPENDIX

Author and Year	Journal	Design	Treatment	Follow-up (months)	Patients (n)	Women (n)	Mean Age (years)	Mean BMI (kg/m2)
Abrahamson et al., 2020 [1]	*J Exp Orthop*	Prospective	Arthroscopy	23.4	135	127	30.0	
					416			
Aguilera-Bohorquez, 2019 [2]	*Arthroscopy*	Retrospective	Arthroscopy	12.0	17	9	34.6	
Aguilera-Bohorquez, 2020 [3]	*Rev Bras Ortop*	Retrospective	Arthroscopy	12.0	34	23	65.5	
					34	23	30.6	
Almasri et al., 2021 [4]	*Knee Surg Sports Traumatol Arthrosc*	Prospective	Arthroscopy	12.0	108	42	36.7	
Alvarez et al., 2010 [5]	*Cartilage*	Retrospective	Arthroscopy		76	58	29.7	
Atzmon et al., 2019 [6]	*J Hip Preserv Surg*	Retrospective	Arthroscopy	60.7	29	13	37.6	
				40.4	35	14	38.1	
Ayeni et al., 2021 [7]	*Am J Sports Med*	Prospective	Arthroscopy	24.0	108	42	36.7	26.9
					106	39	35.4	28.1
Bardakos et al, 2008 [8]	*J Bone Joint Surg Br*	Retrospective	Arthroscopy	12.0	24	10	33.0	
					47	24	35.0	
Basques et al., 2019 [9]	*Am J Sports Med*	Retrospective	Arthroscopy	24.0	389	243	33.0	24.8
					235	164	35.5	25.7
Bech et al., 2021 [10]	*Hip Int*	Prospective	Arthroscopy	12.0	58	35	35.5	23.1
					58	39	33.5	24.2
Beck et al., 2019 [11]	*Am J Sports Med*	Retrospective	Arthroscopy	24.0	112	81	33.6	24.5
					224	153	33.7	24.6
Beck et al., 2020 [12]	*Am J Sports Med*	Retrospective	Arthroscopy	24.0	83	48	39.9	27.2
					166	114	40.0	26.6
Beck et al., 2019 [13]	*Arthroscopy*	Retrospective	Arthroscopy	24.0	335	232	32.8	25.2
Beck et al., 2021 [14]	*Arthroscopy*	Retrospective	Arthroscopy	71.9	25	18	35.0	24.3
				66.7	75	49	35.1	24.0
Beck et al., 2021 [15]	*Arthroscopy*	Retrospective	Arthroscopy	60.0	25		37.4	26.5
					25			

					25			
					25	25		
					25	25		
					25	25		
Bedi et al., 2011 [16]	*Am J Sports Med*	Prospective	Arthroscopy	10.9	10		25.9	
Bellotti et al., 2016 [17]	*Hip Int*	Retrospective	Mini-open	104.4	296			
Bloom et al., 2020 [18]	*Am J Sports Med*	Retrospective	Arthroscopy	24.0	44	44	25.2	23.0
					74	74	37.1	24.8
					76	76	55.2	26.9
Bodendorfer et al., 2021 [19]	*Orthop J Sports Med*	Retrospective	Arthroscopy	24.5	372	231	37.7	24.3
					124	77	38.9	24.4
Bolia et al., 2019 [20]	*Arthroscopy*	Retrospective	Arthroscopy	87.6	42	18	38.0	
				76.8	84	32		
Brick et al., 2020 [21]	*Am J Sports Med*	Prospective	Arthroscopy	24.0	93	57	34.5	25.4
					93	57	34.5	24.3
Briggs et al., 2018 [22]	*Knee Surg Sports Traumatol Arthrosc*	Retrospective	Arthroscopy	61.5	159		40.0	
					71			
Bryan et al., 2016 [23]	*Am J Sports Med*	Prospective	Arthroscopy	51.6	174	138	37.0	
				46.8	27		61.0	
Büchler et al., 2021 [24]	*Clin Orthop Relat Res*	Retrospective	Arthroscopy	132.0	50	45	33.0	21.9
Byrd et al., 2016 [25]	*Arthroscopy*	Retrospective	Arthroscopy	30.0	108	65	15.9	
					122	51	36.8	
Byrd et al., 2019 [26]	*Arthroscopy*		Arthroscopy	18.9	21	10	63.2	
					21	10	35.8	
Byrd, Jones, 2009 [27]	*Arthroscopy*	Prospective	Arthroscopy	120.0	26	13	46.0	
Byrd, Jones, 2009 [28]	*Clin Orthop Relat Res*	Prospective	Arthroscopy	16.0	200	62	33.0	
					200	62	33.4	
Byrd, Jones, 2011 [29]	*Am J Sports Med*	Prospective	Arthroscopy	19.0	200	52	28.6	
Cancienne et al., 2019 [30]	*Am J Sports Med*	Retrospective	Arthroscopy	24.0	40	24	35.5	27.0
					80	49	36.1	24.6
Capogna et al., 2016 [31]	*Arthroscopy*	Prospective	Arthroscopy	26.4	42	34	65.8	26.1

Chahla et al., 2019 [32]	*Am J Sports Med*	Prospective	Arthroscopy	29.5	600	218	32.4	25.0
						169	34.6	25.7
Chahla et al., 2019 [33]	*Am J Sports Med*	Prospective	Arthroscopy	27.8	493	346	34.0	25.0
					92	45	35.4	25.6
					49	26	37.4	26.1
Charles et al., 2021 [34]	*PLoS One*	Retrospective	Arthroscopy	53.0	54	25	35.0	
Cohen et al., 2012 [35]	*Am J Sports Med*	Retrospective	Mini-open	22.0	59	21	32.0	25.5
Comba et al., 2016 [36]	*Muscles Ligaments Tendons J*	Prospective	Arthroscopy	91.0	42	15	38.0	
Cong et al., 2022 [37]	*J Clin Med*	Retrospective	Arthroscopy	10.4	22	14	38.3	
De Lucas Villarrubia, 2021 [38]	*Arthroscopy*	Retrospective	Arthroscopy	29.0	25	6	40.5	
Degen et al., 2017 [39]	*Arthroscopy*	Retrospective	Arthroscopy	36.1	34	16	16.0	
				34.1	296	137	31.0	
Domb et al., 2014 [40]	*Am J Sports Med*	Retrospective	Arthroscopy	26.4	11	4	33.0	24.5
				30.0	22	8	38.8	27.1
Domb et al., 2015 [41]	*Arthroscopy*	Prospective	Arthroscopy	32.8	52	34	54.8	27.3
				33.1	52	34	20.3	22.8
Domb et al., 2017 [42]	*Am J Sports Med*	Retrospective	Arthroscopy	70.0	62	37	41.9	25.5
				72.6	62	37	42.3	26.0
Domb et al., 2020 [43]	*Am J Sports Med*	Prospective	Arthroscopy	50.8	98	62	40.4	25.8
					98	62	40.5	26.1
					98	62	40.7	26.0
Drager et al., 2020 [44]	*Arthroscopy*	Retrospective	Arthroscopy	12.0	173	115	31.3	25.5
					173	134	31.5	25.4
Eberlin et al., 2023 [45]	*Orthop J Sports Med*	Prospective	Arthroscopy	30.4	163	84	37.9	25.9
Essilfie et al., 2020 [46]	*Arthroscopy*	Prospective	Arthroscopy	24.0	84	28	36.0	25.2
					84	56	36.2	24.7
Ezechieli et al., 2016 [47]	*Technology and Health Care*	Prospective	Mini-open	15.0	72	34	32.1	
							28.5	
Fabricant et al., 2015 [48]	*J Bone Joint Surg Am*	Retrospective	Arthroscopy	21.0	37	15	28.0	25.0
					149	75	30.0	24.0
					57	33	29.0	24.0

Feghhi et al., 2020 [49]	*Am J Sports Med*	Retrospective	Arthroscopy	44.4	18	14	28.0	24.0
				26.1	19	14	27.3	22.3
Ferrer-Rivero et al., 2024 [50]	*J ISAKOS*	Retrospective	Arthroscopy	32.4	11	11	32.0	21.7
					22	22		
Filan, Carton, 2020 [51]	*Arthroscopy*	Retrospective	Arthroscopy	27.6	508	80	28.5	
				30.0	458	57	27.6	
Filan, Carton, 2021 [52]	*Arthroscopy*	Retrospective	Arthroscopy	28.8	486		25.9	25.3
Flores et al, 2018 [53]	*Orthop J Sports Med*	Prospective	Arthroscopy	15.5	30	15	37.2	25.6
				13.1	30	13	35.3	25.1
Flores et al., 2020 [54]	*Orthop J Sports Med*	Prospective	Arthroscopy	24.0	72	72	34.2	24.9
					59		35.8	25.5
Foo et al., 2021 [55]	*J Hip Preserv Surg*	Prospective	Arthroscopy	24.0	58	26	36.1	23.1
					48	21	35.9	24.3
Forster-Horvath et al., 2021 [56]	*Arthroscopy*	Retrospective	Arthroscopy	55.0	78		33.1	22.7
Frank et al., 2014 [57]	*Am J Sports Med*	Retrospective	Arthroscopy	29.9	32	20	32.9	25.0
					32	20	32.7	24.8
Frank et al., 2019 [58]	*Orthop J Sports Med*	Retrospective	Arthroscopy	31.2	97	97	36.0	23.8
					97	97	37.8	27.4
Fukui et al., 2015 [59]	*Arthroscopy*	Retrospective	Arthroscopy	40.0	100	51	35.0	25.8
Fukui et al., 2015 [60]	*Bone Joint J*	Retrospective	Arthroscopy	42.0	28	12	34.0	25.6
Gao et al., 2019 [61]	*Chin Med J*	Retrospective	Arthroscopy	22.6	13	7	37.5	
				23.1	229	95	36.2	
Gao et al., 2020 [62]	*J Orthop surg*	Prospective	Arthroscopy	24.0	27	12	57.0	
Gao et al., 2022 [63]	*BMC Musculoskelet Disord*	Retrospective	Arthroscopy	28.1	37	38	36.3	22.2
					34			
Gao et al., 2022 [64]	*J Orthop Surg Res*		Arthroscopy	14.3	194	106	37.1	23.1
Gebhardt et al., 2023 [65]	*Cartilage*	Prospective	Arthroscopy	24.0	11		29.4	24.2
Gedouin et al., 2010 [66]	*Orthop Traumatol Surg Res*	Retrospective	Arthroscopy	15.6	38		36.0	
Gedouin et al., 2010 [67]	*Orthop Traumatol Surg Res*	Prospective	Arthroscopy	10.0	110	32	31.0	
Gicquel et al., 2014 [68]	*Orthop Traumatol Surg Res*	Prospective	Arthroscopy	55.2	51	32		

Giordano et al., 2020 [69]	*Am J Sports Med*	Retrospective	Arthroscopy	75.2	336	210	37.7	25.9
					42			
					9			
					51			
Glaws et al., 2019 [70]	*J Sport Rehabil*	Prospective	Arthroscopy	6.0	16	7	23.4	24.2
					12	8	27.4	25.4
Gupta et al., 2014 [71]	*Am J Sports Med*	Prospective	Arthroscopy	28.3	47	19	37.2	26.1
Gupta et al., 2015 [72]	*Am J Sports Med*	Prospective	Arthroscopy	29.0	595	367	38.0	25.5
Gürsan et al., 2023 [73]	*J Hip Preserv Surg*	Retrospective	Arthroscopy	80.4	52	19	33.9	25.9
Ha et al., 2020 [74]	*J Orthop Surg*	Retrospective	Arthroscopy	24.0	62	6	31.4	23.7
Haefeli et al., 2017 [75]	*Clin Orthop Relat Res*	Retrospective	Arthroscopy	79.0	50	46	35.0	24.0
Hartigan et al., 2017 [76]	*J Hip Preserv Surg*	Retrospective	Arthroscopy	42.0	69	24	43.6	27.1
Hartmann, Gunther, 2009 [77]	*Arch Orthop Trauma Surg*	Retrospective	Arthroscopy	15.0	33	16	31.0	
Hartwell et al., 2021 [78]	*Arthroscopy*	Retrospective	Arthroscopy	89.1	86	61	39.8	24.4
Haskel et al., 2020 [79]	*Am J Sports Med*	Retrospective	Arthroscopy	24.0	38	29	48.2	26.7
					111	84	48.1	26.5
Hassebrock et al., 2019 [80]	*Arthroscopy*	Retrospective	Arthroscopy	63.5	133	86	32.0	24.5
				28.3	133	86	33.0	25.0
Hassebrock et al., 2020 [81]	*Am J Sports Med*	Retrospective	Arthroscopy	26.0	22	6	22.0	26.7
					28	12	19.0	25.4
Hassebrock et al., 2020 [82]	*Am J Sports Med*	Retrospective	Arthroscopy	24.0	49	17	19.4	23.2
					62	18	18.6	24.6
Haviv et al., 2010 [83]	*J Bone Joint Surg Br.*	Retrospective	Arthroscopy	22.0	166	34	32.2	
							35.2	
							43.3	
Haviv, O'Donnell, 2010 [84]	*Orthopedics*	Prospective	Arthroscopy	26.0	82		29.0	
Hevesi et al., 2018 [85]	*Am J Sports Med*	Prospective	Arthroscopy	68.4	48	27	31.8	24.9
					96	49	31.4	24.6
Holleyman et al., 2023 [86]	*Knee Surg Sports Traumatol Arthrosc*	Retrospective	Arthroscopy	12.0	2971	1575	35.8	25.5
					462	343	35.2	25.8
					1530	782	35.3	25.8

Honda et al., 2019 [87]	*Knee Surg Sports Traumatol Arthrosc*	Retrospective	Arthroscopy	32.2	57	26	30.9	22.1
					18	12	56.7	22.5
					9	3	73.5	23.7
Horisberger et al., 2010 [88]	*Arthroscopy*	Prospective	Arthroscopy	36.0	20	4	47.3	
Horisberger et al., 2010 [89]	*Clin Orthop Relat Res*	Prospective	Arthroscopy	27.6	88	28	40.9	
Hufeland et al., 2016 [90]	*Arch Orthop Trauma Surg*	Retrospective	Arthroscopy	66.3	44	20	34.3	
Jimenez et al., 2022 [91]	*Orthop J Sports Med*	Retrospective	Arthroscopy	39.9	20		41.4	30.2
					60		42.5	28.7
Ju et al., 2023 [92]	*Orthop Surg*	Retrospective	Arthroscopy		15	5	37.1	24.3
					26	12	40.2	23.7
Kaplan et al., 2021 [93]	*Arthroscopy*	Retrospective	Arthroscopy	32.4	42	25	39.1	25.8
					19	11	36.0	25.1
Knapik et al., 2020 [94]	*Am J Sports Med*	Retrospective	Arthroscopy	24.0	63	41	31.7	26.0
					45	29	25.2	24.6
Kollmorgen et al., 2024 [95]	*Arthroscopy*	Retrospective	Arthroscopy		25	13	26.5	26.0
					25	14	25.5	26.4
Krych et al., 2013 [96]	*Arthroscopy*	Prospective	Arthroscopy	32.0	18	18	38.0	
					18	18	39.0	
Kunze et al., 2019 [97]	*Am J Sports Med*	Retrospective	Arthroscopy	30.8	250	721	32.3	25.5
					265			
					284			
					295			
Kunze et al., 2019 [98]	*J Hip Preserv Surg*	Retrospective	Arthroscopy	31.2	204	121	34.7	26.3
					102	56	34.9	26.4
Kunze et al., 2020 [99]	*Arthroscopy*	Retrospective	Arthroscopy	60.0	190	115	32.3	24.5
					120	75	36.2	26.4
LaFrance et al., 2015 [100]	*J Hip Preserv Surg*	Prospective	Arthroscopy	18.5	20		34.4	
				23.3	15		34.9	
Lall et al., 2019 [101]	*Arthroscopy*	Retrospective	Arthroscopy	70.6	59	39	37.4	26.9
				71.6	59	39	37.2	26.0
Larson et al., 2012 [102]	*Am J Sports Med*	Prospective	Arthroscopy	42.0	44	17	32.0	

					50	21	28.0	
Larson et al., 2019 [103]	*Arthroscopy*	Retrospective	Arthroscopy	39.8	28	7	15.9	
Larson, Giveans, 2008 [104]	*Arthroscopy*	Prospective	Arthroscopy	9.9	96	42	34.7	
Larson, Giveans, 2009 [105]	*Arthroscopy*	Retrospective	Arthroscopy	21.4	34	9	31.0	
				16.5	37	14	27.0	
Laurito et al., 2021 [106]	*Acta Ortop Bras*	Retrospective	Arthroscopy	17.0	194	63	39.0	
Lee et al., 2019 [107]	*Clin Orthop Surg*	Retrospective	Arthroscopy	92.4	41	20	34.6	24.3
Lee et al., 2021 [108]	*J Hip Preserv Surg*	Retrospective	Arthroscopy	131.5	28	9	36.5	22.7
				135.0	87	27	34.0	22.8
Lee et al., 2022 [109]	*Orthop J Sports Med*	Retrospective	Arthroscopy	38.6	84	62	45.0	26.6
					84	58		28.0
Lee et al., 2023 [110]	*Orthop J Sports Med*	Retrospective	Arthroscopy	24.0	14	3	35.2	24.0
					23	15	41.6	23.8
					38	11	40.2	24.3
Levy et al., 2016 [111]	*Am J Sports Med*	Retrospective	Arthroscopy	24.0	51	29	26.3	23.7
Levy et al., 2017 [112]	*Am J Sports Med*	Retrospective	Arthroscopy	31.2	28	18	35.8	23.8
					56	36	35.2	23.8
Lin et al., 2020 [113]	*Am J Sports Med*	Retrospective	Arthroscopy	74.2	36	25	27.7	24.4
					37	24	41.5	26.3
					36	26	60.2	26.2
Lin et al., 2021 [114]	*Arthroscopy*	Retrospective	Arthroscopy	24.0	152	89	39.2	25.3
					21	18	43.5	27.9
Lindman et al., 2020 [115]	*Am J Sports Med*	Prospective	Arthroscopy	60.0	64	12	24.0	23.7
Lindman et al., 2021 [116]	*Orthop J Sports Med*	Prospective	Arthroscopy	24.0	172	3	28.0	25.6
Litrenta et al., 2019 [117]	*J Pediatr Orthop*	Retrospective	Arthroscopy	50.4	43	38	16.1	20.9
Litrenta et al., 2020 [118]	*J Pediatr Orthop*	Retrospective	Arthroscopy	45.2	69		15.9	21.4
Maimaitimin et al., 2022 [119]	*Orthop J Sports Med*	Retrospective	Arthroscopy	39.4	272	123	37.2	23.4
Maldonado et al., 2018 [120]	*Am J Sports Med*	Retrospective	Arthroscopy	42.5	307	289	27.8	24.7
				43.9	354	277	34.1	25.0
Maldonado et al., 2019 [121]	*Arthroscopy*	Retrospective	Arthroscopy	59.7	18	9	41.2	25.4
				51.4	54	27	41.1	25.3

Maldonado et al., 2019 [122]	*J Hip Preserv Surg*	Retrospective	Arthroscopy	42.4	38	16	43.2	28.3
				54.3	38	16	43.7	26.9
Maldonado et al., 2020 [123]	*Am J Sports Med*	Retrospective	Arthroscopy	72.0	45	34	39.0	25.6
				65.3	45	33	38.3	26.9
Maldonado et al., 2020 [124]	*BMC Musculoskelet Disord*	Retrospective	Arthroscopy	66.8	25	14	41.4	25.1
Mardones et al., 2016 [125]	*Muscles Ligaments Tendons J*	Retrospective	Arthroscopy	48.0	15		33.5	
Marom et al., 2023 [126]	*Knee Surg Sports Traumatol Arthrosc*	Retrospective	Arthroscopy	72.0	119	51	21.6	23.7
Martinez et al., 2019 [127]	*J Orthop*	Prospective	Arthroscopy	6.0	65	25	38.8	23.3
					45	18	37.6	25.4
Martinez et al., 2020 [128]	*Arthrosc Sports Med Rehabil*	Retrospective	Arthroscopy	24.0	76	22	38.8	29.0
					50	2	33.7	4.0
					36	22	36.1	61.2
					18	3	36.8	16.7
Martinez et al., 2023 [129]	*Rev Esp Cir Ortop Traumatol*	Retrospective	Arthroscopy	132.0	17	2	47.8	25.1
					54	9	40.6	25.3
Martinot et al., 2020 [130]	*Orthop Traumatol Surg Res*	Retrospective	Arthroscopy	55.2	55	14	32.3	24.7
			Mini-open		36	6	31.4	24.6
Matsuda et al., 2015 [131]	*J Hip Preserv Surg*	Prospective	Arthroscopy	24.0	127	67	39.8	26.9
					18	6	37.2	28.2
Matsuda et al., 2021 [132]	*Arthroscopy*	Retrospective	Arthroscopy	24.6	16	10	38.0	25.0
					76	15	35.0	26.0
					1301	898	35.0	25.0
May et al., 2020 [133]	*Orthop Traumatol Surg Res*	Prospective	Arthroscopy	14.6	42	14	35.4	24.4
				15.9	79	58	32.8	21.8
					66			
McConkey et al., 2019 [134]	*J Pediatr Orthop*	Prospective	Arthroscopy	24.0	12	5	15.7	20.3
					12	5	16.5	21.5
Menge et al., 2021 [135]	*Am J Sports Med*	Prospective	Arthroscopy	134.0	60		16.0	22.0
						49	16.0	21.0
Mercier et al., 2019 [136]	*Orthop Traumatol Surg Res*	Retrospective	Arthroscopy	29.6	20	6	32.5	23.8

				31.4	23	5	33.5	23.5
Migliorini et al., 2023 [137]	*J Orthop Surg Res*		Arthroscopy	72.8	108	46	41.5	27.0
Mihalic et al., 2021 [138]	*Int Orthop*	Retrospective	Mini-open	50.0	5		37.0	31.1
Mohan et al., 2017 [139]	*Arthroscopy*	Retrospective	Arthroscopy	34.0	50	33	17.8	23.1
Moon et al., 2020 [140]	*Arthroscopy*	Retrospective	Arthroscopy	62.4	73	15	34.4	24.1
Moriya et al., 2017 [141]	*J Orthop Surg Res*	Retrospective	Arthroscopy	28.0	23	17	59.3	
Mortensen et al., 2023 [142]	*Arthrosc Sports Med Rehabil*	Prospective	Arthroscopy		57	32	31.2	25.2
Mullins et al., 2019 [143]	*Knee Surg Sports Traumatol Arthrosc*	Prospective	Arthroscopy	12.0	47		24.6	
					32		24.3	
Mullins et al., 2021 [144]	*Orthop J Sports Med*	Prospective	Arthroscopy	27.1	760	50	26.3	
Nakashima et al., 2019 [145]	*Am J Sports Med*	Retrospective	Arthroscopy	37.0	25	7	52.6	24.3
				31.9	126	73	36.5	21.8
Nakashima et al., 2021 [146]	*Clin J Sport Med*	Retrospective	Arthroscopy	35.1	79	45	51.3	22.6
				29.7	18	9	56.3	24.1
Nawabi et al., 2016 [147]	*Am J Sports Med*	Prospective	Arthroscopy	31.3	46	22	29.8	
					131	73	29.6	
Nguyen et al., 2020 [148]	*Am J Sports Med*	Prospective	Arthroscopy	24.0	166	91	35.3	25.1
Nho et al., 2011 [149]	*Am J Sports Med*	Retrospective	Arthroscopy	27.0	47	13	22.8	
					47	13	22.8	
Nielsen et al., 2014 [150]	*BMC Musculoskelet Disord*	Prospective	Arthroscopy	24.0	117	69	37.0	
Nwachukwu et al., 2020 [151]	*Am J Sports Med*	Prospective	Arthroscopy	60.0	283	179	34.2	26.1
Öhlin et al., 2019 [152]	*Knee Surg Sports Traumatol Arthrosc*	Prospective	Arthroscopy	60.0	184	74	38.0	
Ohlsen et al., 2024 [153]	*Orthop J Sports Med*	Retrospective	Arthroscopy	13.9	22	21	31.1	25.2
					22	21	27.8	26.0
Olivero et al., 2023 [154]	*SICOT-J*	Retrospective	Arthroscopy	12.0	54	24	35.0	
			Mini-open		44	12	39.9	
O'Reilly et al., 2022 [155]	*Arthrosc Sports Med Rehabil*	Retrospective	Arthroscopy	29.3	104	104	26.9	23.0
				30.0	56		23.0	25.5
Ortiz-Declet et al., 2019 [156]	*Arthroscopy*	Retrospective	Arthroscopy	47.4	34	19	20.8	22.8
Owens et al., 2022 [157]	*Orthop J Sports Med*	2022	Arthroscopy	36.1	30	16	29.4	25.9

				34.5	60	33	27.5	24.8
Owens et al., 2022 [158]	*Orthop J Sports Med*	2022	Arthroscopy	30.2	29		40.3	27.8
				27.6	29	29	40.5	27.1
Özbek et al., 2021 [159]	*Acta Orthop Traumatol Turc*	Prospective	Arthroscopy	24.0	34	20	32.3	24.9
Palmer et al., 2012 [160]	*Arthroscopy*	Retrospective	Arthroscopy	46.0	185	102	40.2	
Pansard et al., 2021 [161]	*Orthop Traumatol Surg Res*	Prospective	Arthroscopy	12.0	42	74	33.3	23.6
					10			
					145			
Parvaresh et al., 2020 [162]	*Am J Sports Med*	Retrospective	Arthroscopy	62.1	60	33	37.2	22.5
					40	22	37.3	27.0
					20	11	38.3	31.6
					20	11	37.7	39.4
Parvaresh et al., 2021 [163]	*Am J Sports Med*	Retrospective	Arthroscopy	24.0	329	218	32.5	24.0
					329	218	32.6	24.0
Parvaresh et al., 2021 [164]	*Phys Ther Sport*	Retrospective	Arthroscopy	24.0	23	14	36.2	22.8
Perets et al., 2017 [165]	*Arthroscopy*	Prospective	Arthroscopy	35.7	10	10	14.7	21.7
Perets et al., 2018 [166]	*J Bone Joint Surg Am*	Retrospective	Arthroscopy	71.6	74	45	44.2	34.3
				71.3	74	45		22.7
Perets et al., 2019 [167]	*Arthroscopy*	Retrospective	Arthroscopy	69.3	57	50	26.5	23.0
				71.7	57	50	28.0	23.1
Perets et al., 2019 [168]	*J Am Acad Orthop Surg*	Retrospective	Arthroscopy	68.7	407	219	32.4	24.6
Philippon et al., 2007 [169]	*Knee Surg Sports Traumatol Arthrosc*	Retrospective	Arthroscopy	19.2	45	3	31.0	
Philippon et al., 2009 [170]	*J Bone Joint Surg Br*	Prospective	Arthroscopy	27.6	112	62	40.6	24.0
Philippon et al., 2010 [171]	*Am J Sports Med*	Retrospective	Arthroscopy	24.0	28		27.0	
Philippon et al., 2012 [172]	*Arthroscopy*	Retrospective	Arthroscopy	37.5	153	81	57.0	
Philippon et al., 2012 [173]	*Arthroscopy*	Retrospective	Arthroscopy	36.0	60	41	15.0	21.1
Polesello et al., 2009 [174]	*Rev Bras Ortop*	Retrospective	Arthroscopy	27.0	28	9	34.0	
Polesello et al., 2014 [175]	*Hip Int*	Retrospective	Arthroscopy	73.2	24	3	34.6	
Pontiff et al., 2016 [176]	*Int J Sports Phys Ther*	Retrospective	Arthroscopy	6.0	35	35	25.7	
					131	131	33.5	
Rafols et al., 2015 [177]	*Arthroscopy*	Prospective	Arthroscopy	24.0	30		35.3	

					27			
Ramos et al., 2020 [178]	*J Hip Preserv Surg*	Retrospective	Arthroscopy	19.2	10		45.0	
Rhee et al., 2016 [179]	*Arch Orthop Trauma Surg*	Prospective	Arthroscopy	32.3	19	9	33.8	22.4
				31.8	18	13	34.6	22.1
Riedl et al., 2020 [180]	*J Clin Med*	Retrospective	Arthroscopy	33.4	14	7	36.2	23.1
				26.3	26	10	36.1	25.1
Rivera et al., 2019 [181]	*J Invest Surg*	Retrospective	Arthroscopy	24.0	40	13	41.8	
					40	14	42.5	
Rosinsky et al., 2019 [182]	*Am J Sports Med*	Retrospective	Arthroscopy	35.0	44	19	17.3	23.3
					38	30		23.7
Said et al., 2019 [183]	*SICOT-J*	Retrospective	Arthroscopy	32.8	85	13	30.3	
Saito et al., 2019 [184]	*Am J Sports Med*	Retrospective	Arthroscopy	24.0	25		17.5	22.2
							22.0	22.4
							18.5	22.0
							17.0	22.4
Saks et al., 2021 [185]	*Arthroscopy*	Retrospective	Arthroscopy	63.6	41	27	38.9	26.8
				65.6	41	25	37.2	25.5
Sanders et al., 2016 [186]	*Knee Surg Sports Traumatol Arthrosc*	Retrospective	Arthroscopy	30.0	46	31	42.4	
Sansone et al., 2015 [187]	*J Hip Preserv Surg*	Prospective	Arthroscopy	12.8	75	18	47.0	
Sansone et al., 2015 [188]	*Orthop J Sports Med*	Retrospective	Arthroscopy	12.3	85	21	25.0	
Sansone et al., 2016 [189]	*Scand J Med Sci Sports*	Prospective	Arthroscopy	25.4	289	99	37.0	
Scanaliato et al., 2020 [190]	*Arthroscopy*	Retrospective	Arthroscopy	24.3	30	17	30.4	23.4
Schilders et al., 2011 [191]	*J Bone Joint Surg Br*	Retrospective	Arthroscopy	24.0	96	25	37.0	
Seijas et al., 2023 [192]	*Surg J (N Y)*	Retrospective	Arthroscopy	24.0	15	2	42.7	24.4
					50	16	39.4	23.9
Shao et al., 2022 [193]	*Orthop Surg*	Retrospective	Arthroscopy	39.1	33	15	39.3	24.3
				38.6	167	82	37.4	23.3
Shuang et al., 2024 [194]	*Orthop J Sports Med*	Retrospective	Arthroscopy	24.0	63	56	37.0	23.5

Singh et al., 2010 [195]	*Arthroscopy*	Prospective	Arthroscopy	22.0	24		22.0	24.0
Skendzel et al., 2014 [196]	*Am J Sports Med*	Prospective	Arthroscopy	73.0	383		37.0	
					63		46.0	
Skowronek et al., 2017 [197]	*Indian J Orthop*	Retrospective	Mini-open	45.0	39	14	29.3	
Snaebjörnsson et al., 2022 [198]	*Arthroscopy*	Prospective	Arthroscopy	24.0	84	17	19.8	
Sobti et al., 2020 [199]	*J Hip Preserv Surg*	Retrospective	Arthroscopy	12.0	46	27	45.0	
					65	40	45.0	
Soriano et al., 2021 [200]	*Arthroscopy*	Retrospective	Arthroscopy	24.0	37	19	36.8	25.9
					37	19	36.7	26.0
Stake et al., 2013 [201]	*Am J Sports Med*	Prospective	Arthroscopy	24.0	20	3	39.0	
					20	3		
Stone et al., 2019 [202]	*Am J Sports Med*	Retrospective	Arthroscopy	24.0	514	334	32.4	25.2
					174	115	35.9	25.9
Stone et al., 2019 [203]	*Arthroscopy*	Retrospective	Arthroscopy	24.0	464	437	31.6	24.6
					60			
					47			
					10			
Stone et al., 2019 [204]	*J Hip Preserv Surg*	Prospective	Arthroscopy	29.3	100	100	22.7	23.1
					25	25	18.0	22.4
Sugarman et al., 2021 [205]	*Orthop J Sports Med*	Prospective	Arthroscopy	24.0	28	8	33.7	25.7
					28	22	45.0	24.4
Sutton et al., 2021 [206]	*Clin Orthop Relat Res*	Retrospective	Mini-open	48.0	60	43	27.0	26.0
					20	12	27.0	28.0
Thaunat et al., 2020 [207]	*Orthop Traumatol Surg Res*	Retrospective	Arthroscopy	34.7	39	9	28.8	
					25	4	28.5	
Tjong et al., 2016 [208]	*Orthop J Sports Med*	Retrospective	Arthroscopy	24.0	13	7	44.0	
					10	8	43.7	
Torabian et al., 2023 [209]	*Am J Sports Med*	Retrospective	Arthroscopy	38.2	70	37	39.4	26.1
				39.2	87	40	35.8	25.1
Torabian et al., 2024 [210]	*Am J Sports Med*	Retrospective	Arthroscopy	23.3	28	18	36.4	24.2
				24.9	31	15	41.5	25.8

				21.4	15	9	35.8	26.4
Tran et al., 2013 [211]	*ANZ J Surg*	Retrospective	Arthroscopy	14.0	34	5	15.7	
Ukwuani et al., 2018 [212]	*Arthroscopy*	Retrospective	Arthroscopy	23.0	21	62	19.9	21.9
					43		23.3	23.2
Vahedi et al., 2019 [213]	*J Arthroplasty*	Retrospective	Mini-open	51.6	73	35	30.7	
				49.2	550	268	34.5	
Vahedi et al., 2019 [214]	*J Arthroplasty*	Retrospective	Mini-open	57.6	51	33	27.4	
				49.2	550	253	34.5	
Varshneya et al., 2022 [215]	*Arthroscopy*	Retrospective	Arthroscopy	12.0	2564	1969	36.7	
					11245	6882	36.2	
Wang et al., 2011 [216]	*Orthop Surg*	Retrospective	Arthroscopy	11.6	21	12	37.1	
Wang et al., 2023 [217]	*Orthop J Sports Med*	Prospective	Arthroscopy	112.8	32	24	27.7	26.6
Weber et al., 2020 [218]	*Orthop J Sports Med*	Retrospective	Arthroscopy		1	2	19.5	
					15	2		
					8	1		
					6			
					9	6		
White et al., 2020 [219]	*Arthroscopy*	Retrospective	Arthroscopy	45.6	136	130	48.1	
				67.2	82	76	47.0	
				43.2	94	87	34.6	
White et al., 2022 [220]	*Arthroscopy*	Prospective	Arthroscopy	46.2	47	36	42.2	
Winge et al., 2021 [221]	*J Hip Preserv Surg*	Retrospective	Arthroscopy	80.4	29	18	16.3	
Wirries et al., 2021 [222]	*Int Orthop*	Retrospective	Mini-open	51.6	47		43.7	27.4
					32		46.2	27.3
Yacovelli et al., 2021 [223]	*Clin Orthop Relat Res*	Retrospective	Mini-open	60.0	130	70	47.0	
					260	133	28.0	
Yang et al., 2022 [224]	*Orthop J Sports Med*	Retrospective	Arthroscopy	38.4	80	49	36.5	22.8
				38.7	80	49	35.4	22.7
				37.5	81	49	35.6	22.5
				38.5	81	51	37.7	22.9

				38.6	80	49	37.6	23.1
				38.2	80	50	37.1	22.9
Yang et al., 2023 [225]	*Orthop Surg*	Retrospective	Arthroscopy	38.2	57	27	53.2	24.4
Yang et al., 2023 [226]	*Orthop Surg*	Retrospective	Arthroscopy	40.0	71	22	34.6	23.7
				39.6	71	24	35.2	24.0
Yin et al., 2021 [227]	*Orthop Surg*	Retrospective	Arthroscopy	16.0	26	14	38.2	24.7
				18.0	30	17	40.1	24.1
You et al., 2020 [228]	*Orthop J Sports Med*	Prospective	Arthroscopy	24.0	168	91	35.3	
Zacharias et al., 2024 [229]	*Arthrosc Sports Med Rehabil*	Arthroscopy	Arthroscopy	12.0	21	13	35.6	24.0
					79	54	34.2	25.8
Zhang et al., 2021 [230]	*Arthroscopy*	Retrospective	Arthroscopy	24.0	16	6	32.4	21.8
					11	4	34.0	23.1
Zimmerer et al., 2020 [231]	*Orthop J Sports Med*	Retrospective	Arthroscopy	43.8	8	24	35.9	25.0
					7			
					6			
					15			
Zimmerer et al., 2021 [232]	*Arthroscopy*	Retrospective	Arthroscopy	132.7	51	21	43.0	22.0
				131.2	61	20	44.1	22.4
Zimmerer et al., 2021 [233]	*Orthop J Sports Med*	Prospective	Arthroscopy	132.0	44	17	42.2	22.3

Table 1. Generalities of the included studies.

1. Abrahamson J, Lindman I, Sansone M, Öhlin A, Jonasson P, Karlsson J, Baranto A (2020) Low rate of high-level athletes maintained a return to pre-injury sports two years after arthroscopic treatment for femoroacetabular impingement syndrome. J Exp Orthop 7 (1):44. doi:10.1186/s40634-020-00263-5
2. Aguilera-Bohorquez B, Brugiatti M, Coaquira R, Cantor E (2019) Frequency of Subspine Impingement in Patients With Femoroacetabular Impingement Evaluated With a 3-Dimensional Dynamic Study. Arthroscopy 35 (1):91-96. doi:10.1016/j.arthro.2018.08.035
3. Aguilera-Bohórquez B, Brugiatti M, Coaquira R, Cardozo O, Cantor E (2020) Functional Outcomes of Arthroscopic Treatment in Femoroacetabular Impingement in Patients over 60 Years Old Compared with Patients Aged 40 Years or Younger. Rev Bras Ortop (Sao Paulo) 55 (6):715-721. doi:10.1055/s-0040-1708515
4. Almasri M, Simunovic N, Heels-Ansdell D, Ayeni OR (2021) Femoroacetabular impingement surgery leads to early pain relief but minimal functional gains past 6 months: experience from the FIRST trial. Knee Surg Sports Traumatol Arthrosc 29 (5):1362-1369. doi:10.1007/s00167-020-06401-x
5. Alvarez DR, Mardones RM (2010) Efficacy of Osseous Abnormalities Correction with Arthroscopic Surgery in Femoroacetabular Impingement. Cartilage 1 (3):233-237. doi:10.1177/1947603510362252
6. Atzmon R, Sharfman ZT, Haviv B, Frankl M, Rotem G, Amar E, Drexler M, Rath E (2019) Does capsular closure influence patient-reported outcomes in hip arthroscopy for femoroacetabular impingement and labral tear? J Hip Preserv Surg 6 (3):199-206. doi:10.1093/jhps/hnz025
7. Ayeni OR, Karlsson J, Heels-Ansdell D, Thabane L, Musahl V, Simunovic N, Duong A, Bhandari M, Bedi A, Järvinen T, Naudie D, Seppänen M, Slobogean G, Skelly M, Shanmugaraj A, Crouch S, Sprague S, Buckingham L, Ramsay T, Lee J, Kousa P, Carsen S, Choudur H, Sim Y, Johnston K, Sprague S, Wong I, Murphy R, Sparavalo S, Whelan D, Khan R, Wood GCA, Howells F, Grant H, Naudie D, Zomar B, Pollock M, Willits K, Firth A, Wanlin S, Remtulla A, Kaniki N, Belzile EL, Turmel S, Jørgensen U, Gam-Pedersen A, Hatanpää T, Sihvonen R, Raivio M, Toivonen P, Routapohja MP (2021) Osteochondroplasty and Labral Repair for the Treatment of Young Adults With Femoroacetabular Impingement: A Randomized Controlled Trial. Am J Sports Med 49 (1):25-34. doi:10.1177/0363546520952804
8. Bardakos NV, Vasconcelos JC, Villar RN (2008) Early outcome of hip arthroscopy for femoroacetabular impingement: the role of femoral osteoplasty in symptomatic improvement. J Bone Joint Surg Br 90 (12):1570-1575. doi:10.1302/0301-620x.90b12.21012
9. Basques BA, Waterman BR, Ukwuani G, Beck EC, Neal WH, Friel NA, Stone AV, Nho SJ (2019) Preoperative Symptom Duration Is Associated With Outcomes After Hip Arthroscopy. Am J Sports Med 47 (1):131-137. doi:10.1177/0363546518808046
10. Bech NH, Sierevelt IN, de Waard S, Joling BSH, Kerkhoffs G, Haverkamp D (2023) Capsular closure versus unrepaired interportal capsulotomy after hip arthroscopy in patients with femoroacetabular impingement: results of a patient-blinded randomised controlled trial. Hip Int 33 (1):94-101. doi:10.1177/11207000211005762
11. Beck EC, Nwachukwu BU, Chahla J, Jan K, Keating TC, Suppauksorn S, Nho SJ (2019) Patients With Borderline Hip Dysplasia Achieve Clinically Significant Outcome After Arthroscopic Femoroacetabular Impingement Surgery: A Case-Control Study With Minimum 2-Year Follow-up. Am J Sports Med 47 (11):2636-2645. doi:10.1177/0363546519865919
12. Beck EC, Nwachukwu BU, Chapman R, Gowd AK, Waterman BR, Nho SJ (2020) The Influence of Lumbosacral Spine Pathology on Minimum 2-Year Outcome After Hip Arthroscopy: A Nested Case-Control Analysis. Am J Sports Med 48 (2):403-408. doi:10.1177/0363546519892916
13. Beck EC, Nwachukwu BU, Kunze KN, Chahla J, Nho SJ (2019) How Can We Define Clinically Important Improvement in Pain Scores After Hip Arthroscopy for Femoroacetabular Impingement Syndrome? Minimum 2-Year Follow-up Study. Am J Sports Med 47 (13):3133-3140. doi:10.1177/0363546519877861
14. Beck EC, Nwachukwu BU, Chahla J, Clapp IM, Jan K, Nho SJ (2021) Complete Capsular Closure Provides Higher Rates of Clinically Significant Outcome Improvement and Higher Survivorship Versus Partial Closure After Hip Arthroscopy at Minimum 5-Year Follow-Up.

Arthroscopy 37 (6):1833-1842. doi:10.1016/j.arthro.2021.01.035
15. Beck EC, Drager J, Nwachukwu BU, Jan K, Rasio J, Nho SJ (2021) Gender and Age-Specific Differences Observed in Rates of Achieving Meaningful Clinical Outcomes 5-Years After Hip Arthroscopy for Femoroacetabular Impingement Syndrome. Arthroscopy 37 (8):2488-2496.e2481. doi:10.1016/j.arthro.2021.02.033
16. Bedi A, Dolan M, Hetsroni I, Magennis E, Lipman J, Buly R, Kelly BT (2011) Surgical treatment of femoroacetabular impingement improves hip kinematics: a computer-assisted model. Am J Sports Med 39 Suppl:43s-49s. doi:10.1177/0363546511414635
17. Bellotti V, Cardenas C, Astarita E, Moya E, De Meo F, Ezechieli M, Ribas M (2016) Mini-open approach for femoroacetabular impingement: 10 years experience and evolved indications. Hip Int 26 Suppl 1:38-42. doi:10.5301/hipint.5000408
18. Bloom DA, Fried JW, Bi AS, Kaplan DJ, Chintalapudi N, Youm T (2020) Age-Associated Pathology and Functional Outcomes After Hip Arthroscopy in Female Patients: Analysis With 2-Year Follow-up. Am J Sports Med 48 (13):3265-3271. doi:10.1177/0363546520957712
19. Bodendorfer BM, Alter TD, DeFroda SF, Wolff AB, Carreira DS, Cristoforetti JJ, Matsuda DK, Salvo JP, Kivlan BR, Nho SJ (2021) Multicenter Outcomes After Hip Arthroscopy: Comparative Analysis of Patients Undergoing Concomitant Labral Repair and Ligamentum Teres Debridement Versus Isolated Labral Repair. Orthop J Sports Med 9 (9):23259671211036469. doi:10.1177/23259671211036469
20. Bolia IK, Fagotti L, Briggs KK, Philippon MJ (2019) Midterm Outcomes Following Repair of Capsulotomy Versus Nonrepair in Patients Undergoing Hip Arthroscopy for Femoroacetabular Impingement With Labral Repair. Arthroscopy 35 (6):1828-1834. doi:10.1016/j.arthro.2019.01.033
21. Brick CR, Bacon CJ, Brick MJ (2020) Importance of Retaining Sufficient Acetabular Depth: Successful 2-Year Outcomes of Hip Arthroscopy for Patients With Pincer Morphology as Compared With Matched Controls. Am J Sports Med 48 (10):2471-2480. doi:10.1177/0363546520937301
22. Briggs KK, Soares E, Bhatia S, Philippon MJ (2019) Postoperative alpha angle not associated with patient-centered midterm outcomes following hip arthroscopy for FAI. Knee Surg Sports Traumatol Arthrosc 27 (10):3105-3109. doi:10.1007/s00167-018-4933-3
23. Bryan AJ, Krych AJ, Pareek A, Reardon PJ, Berardelli R, Levy BA (2016) Are Short-term Outcomes of Hip Arthroscopy in Patients 55 Years and Older Inferior to Those in Younger Patients? Am J Sports Med 44 (10):2526-2530. doi:10.1177/0363546516652114
24. Büchler L, Grob V, Anwander H, Lerch TD, Haefeli PC (2021) Good Outcome Scores and Low Conversion Rate to THA 10 Years After Hip Arthroscopy for the Treatment of Femoroacetabular Impingement. Clin Orthop Relat Res 479 (10):2256-2264. doi:10.1097/corr.0000000000001778
25. Byrd JW, Jones KS, Gwathmey FW (2016) Arthroscopic Management of Femoroacetabular Impingement in Adolescents. Arthroscopy 32 (9):1800-1806. doi:10.1016/j.arthro.2016.02.019
26. Byrd JWT, Jones KS (2019) Arthroscopic Acetabular Labral Repair in Patients Over the Age of 60 Years: A Matched Case-Control Study. Arthroscopy 35 (5):1406-1410. doi:10.1016/j.arthro.2018.11.015
27. Byrd JW, Jones KS (2009) Hip arthroscopy for labral pathology: prospective analysis with 10-year follow-up. Arthroscopy 25 (4):365-368. doi:10.1016/j.arthro.2009.02.001
28. Byrd JW, Jones KS (2009) Arthroscopic femoroplasty in the management of cam-type femoroacetabular impingement. Clin Orthop Relat Res 467 (3):739-746. doi:10.1007/s11999-008-0659-8
29. Byrd JW, Jones KS (2011) Arthroscopic management of femoroacetabular impingement in athletes. Am J Sports Med 39 Suppl:7s-13s. doi:10.1177/0363546511404144
30. Cancienne J, Kunze KN, Beck EC, Chahla J, Suppauksorn S, Nho SJ (2019) Influence of Cigarette Smoking at the Time of Surgery on Postoperative Outcomes in Patients With Femoroacetabular Impingement: A Matched-Pair Cohort Analysis. Am J Sports Med 47 (5):1138-1144. doi:10.1177/0363546519832545
31. Capogna BM, Ryan MK, Begly JP, Chenard KE, Mahure SA, Youm T (2016) Clinical Outcomes of Hip Arthroscopy in Patients 60 or Older: A

Minimum of 2-Year Follow-up. Arthroscopy 32 (12):2505-2510. doi:10.1016/j.arthro.2016.06.026
32. Chahla J, Nwachukwu BU, Beck EC, Neal WH, Cancienne J, Okoroha KR, Ahn J, Nho SJ (2019) Influence of Acetabular Labral Tear Length on Outcomes After Hip Arthroscopy for Femoroacetabular Impingement Syndrome With Capsular Plication. Am J Sports Med 47 (5):1145-1150. doi:10.1177/0363546519831291
33. Chahla J, Beck EC, Okoroha K, Cancienne JM, Kunze KN, Nho SJ (2019) Prevalence and Clinical Implications of Chondral Injuries After Hip Arthroscopic Surgery for Femoroacetabular Impingement Syndrome. Am J Sports Med 47 (11):2626-2635. doi:10.1177/0363546519865912
34. Charles T, Jayankura M (2021) Evaluation of hip arthroscopy using a hip-specific distractor for the treatment of femoroacetabular impingement. PLoS One 16 (2):e0246655. doi:10.1371/journal.pone.0246655
35. Cohen SB, Huang R, Ciccotti MG, Dodson CC, Parvizi J (2012) Treatment of femoroacetabular impingement in athletes using a mini-direct anterior approach. Am J Sports Med 40 (7):1620-1627. doi:10.1177/0363546512445883
36. Comba F, Yacuzzi C, Ali PJ, Zanotti G, Buttaro M, Piccaluga F (2016) Joint preservation after hip arthroscopy in patients with FAI. Prospective analysis with a minimum follow-up of seven years. Muscles Ligaments Tendons J 6 (3):317-323. doi:10.11138/mltj/2016.6.3.317
37. Cong S, Pan J, Huang G, Xie D, Zeng C (2022) The Modified Longitudinal Capsulotomy by Outside-In Approach in Hip Arthroscopy for Femoroplasty and Acetabular Labrum Repair-A Cohort Study. J Clin Med 11 (15). doi:10.3390/jcm11154548
38. De Lucas Villarrubia JC, Méndez Alonso M, Sanz Pérez MI, Trell Lesmes F, Panadero Tapia A (2022) Acellular Matrix-Induced Chondrogenesis Technique Improves the Results of Chondral Lesions Associated With Femoroacetabular Impingement. Arthroscopy 38 (4):1166-1178. doi:10.1016/j.arthro.2021.08.022
39. Degen RM, Mayer SW, Fields KG, Coleman SH, Kelly BT, Nawabi DH (2017) Functional Outcomes and Cam Recurrence After Arthroscopic Treatment of Femoroacetabular Impingement in Adolescents. Arthroscopy 33 (7):1361-1369. doi:10.1016/j.arthro.2017.01.044
40. Domb BG, El Bitar YF, Stake CE, Trenga AP, Jackson TJ, Lindner D (2014) Arthroscopic labral reconstruction is superior to segmental resection for irreparable labral tears in the hip: a matched-pair controlled study with minimum 2-year follow-up. Am J Sports Med 42 (1):122-130. doi:10.1177/0363546513508256
41. Domb BG, Linder D, Finley Z, Botser IB, Chen A, Williamson J, Gupta A (2015) Outcomes of hip arthroscopy in patients aged 50 years or older compared with a matched-pair control of patients aged 30 years or younger. Arthroscopy 31 (2):231-238. doi:10.1016/j.arthro.2014.08.030
42. Domb BG, Chaharbakhshi EO, Rybalko D, Close MR, Litrenta J, Perets I (2017) Outcomes of Hip Arthroscopic Surgery in Patients With Tönnis Grade 1 Osteoarthritis at a Minimum 5-Year Follow-up: A Matched-Pair Comparison With a Tönnis Grade 0 Control Group. Am J Sports Med 45 (10):2294-2302. doi:10.1177/0363546517706957
43. Domb BG, Annin S, Chen JW, Kyin C, Rosinsky PJ, Maldonado DR, Meghpara MB, Lall AC, Shapira J (2020) Optimal Treatment of Cam Morphology May Change the Natural History of Femoroacetabular Impingement. Am J Sports Med 48 (12):2887-2896. doi:10.1177/0363546520949541
44. Drager J, Rasio J, Newhouse A, Beck E, Chahla J, Nho SJ (2020) Patients With a Hypotrophic Labrum Achieve Similar Outcomes After Primary Labral Repair Compared With Patients With a Normal-Sized Labrum: A Matched Cohort Analysis of 346 Patients With Femoroacetabular Impingement Syndrome. Arthroscopy 36 (10):2614-2620. doi:10.1016/j.arthro.2020.05.039
45. Eberlin CT, Kucharik MP, Abraham PF, Nazal MR, Conaway WK, Varady NH, Martin SD (2023) Puncture Capsulotomy Technique for Hip Arthroscopy: Midterm Functional Outcomes. Orthop J Sports Med 11 (1):23259671221144056. doi:10.1177/23259671221144056
46. Essilfie AA, Bloom DA, Zusmanovich M, Kester B, Wolfson T, Youm T (2020) Staged Bilateral Hip Arthroscopy Compared With a Matched Unilateral Hip Arthroscopy Group: Minimum 2-Year Follow-Up. Arthroscopy 36 (7):1856-1861. doi:10.1016/j.arthro.2020.02.025
47. Ezechieli M, De Meo F, Bellotti V, Cardenas C, Astarita E, Cavaliere P, Windhagen H, Ribas M (2016) Arthroscopic assisted mini-open

approach of the hip: Early multicentric experience. Technol Health Care 24 (3):359-365. doi:10.3233/thc-151127
48. Fabricant PD, Fields KG, Taylor SA, Magennis E, Bedi A, Kelly BT (2015) The effect of femoral and acetabular version on clinical outcomes after arthroscopic femoroacetabular impingement surgery. J Bone Joint Surg Am 97 (7):537-543. doi:10.2106/jbjs.N.00266
49. Feghhi D, Shearin J, Bharam S (2020) Arthroscopic Management of Subspinous Impingement in Borderline Hip Dysplasia and Outcomes Compared With a Matched Cohort With Nondysplastic Femoroacetabular Impingement. Am J Sports Med 48 (12):2919-2926. doi:10.1177/0363546520951202
50. Ferrer-Rivero J, Chahla J, Lizano-Diez X, Andriola V, López-Zabala I, Soler-Cano A, Tey-Pons M (2024) Hip arthroscopy is an effective treatment for high-level female athletes. J isakos. doi:10.1016/j.jisako.2024.02.010
51. Filan D, Carton P (2020) Routine Interportal Capsular Repair Does Not Lead to Superior Clinical Outcome Following Arthroscopic Femoroacetabular Impingement Correction With Labral Repair. Arthroscopy 36 (5):1323-1334. doi:10.1016/j.arthro.2019.12.002
52. Filan D, Carton P (2021) Chronic Hip Injury Has a Negative Emotional Impact on the Male Athlete With Femoroacetabular Impingement. Arthroscopy 37 (2):566-576. doi:10.1016/j.arthro.2020.10.035
53. Flores SE, Borak KR, Zhang AL (2018) Hip Arthroscopic Surgery for Femoroacetabular Impingement: A Prospective Analysis of the Relationship Between Surgeon Experience and Patient Outcomes. Orthop J Sports Med 6 (2):2325967118755048. doi:10.1177/2325967118755048
54. Flores SE, Chambers CC, Borak KR, Zhang AL (2020) Is There a Gender Gap in Outcomes After Hip Arthroscopy for Femoroacetabular Impingement? Assessment of Clinically Meaningful Improvements in a Prospective Cohort. Orthop J Sports Med 8 (7):2325967119900561. doi:10.1177/2325967119900561
55. Foo GL, Knudsen JS, Bacon CJ, Mei-Dan O, McConkey MO, Brick MJ (2021) Peri-operative platelet-rich plasma in arthroscopic femoroacetabular impingement surgery: a randomized controlled trial. J Hip Preserv Surg 8 (1):14-21. doi:10.1093/jhps/hnab001
56. Forster-Horváth C, Unterreithmeier U, Fries S, Ganal S, Gütler J, Vogel N, Herzog RF (2021) Midterm Follow-Up and Assessment of Cartilage Thickness by Arthro-Magnetic Resonance Imaging After Arthroscopic Cam Resection, Labral Repair, and Rim Trimming Without Labral Detachment. Arthroscopy 37 (2):541-551. doi:10.1016/j.arthro.2020.10.012
57. Frank RM, Lee S, Bush-Joseph CA, Kelly BT, Salata MJ, Nho SJ (2014) Improved outcomes after hip arthroscopic surgery in patients undergoing T-capsulotomy with complete repair versus partial repair for femoroacetabular impingement: a comparative matched-pair analysis. Am J Sports Med 42 (11):2634-2642. doi:10.1177/0363546514548017
58. Frank RM, Kunze KN, Beck EC, Neal WH, Bush-Joseph CA, Nho SJ (2019) Do Female Athletes Return to Sports After Hip Preservation Surgery for Femoroacetabular Impingement Syndrome?: A Comparative Analysis. Orthop J Sports Med 7 (3):2325967119831758. doi:10.1177/2325967119831758
59. Fukui K, Briggs KK, Trindade CA, Philippon MJ (2015) Outcomes After Labral Repair in Patients With Femoroacetabular Impingement and Borderline Dysplasia. Arthroscopy 31 (12):2371-2379. doi:10.1016/j.arthro.2015.06.028
60. Fukui K, Trindade CA, Briggs KK, Philippon MJ (2015) Arthroscopy of the hip for patients with mild to moderate developmental dysplasia of the hip and femoroacetabular impingement: Outcomes following hip arthroscopy for treatment of chondrolabral damage. Bone Joint J 97-b (10):1316-1321. doi:10.1302/0301-620x.97b10.35303
61. Gao GY, Zhang X, Dai LH, Huang HJ, Wu RQ, Ju XD, Mei Y, Niu XY, Wang JQ, Xu Y (2019) Heterotopic ossification after arthroscopy for hip impingement syndrome. Chin Med J (Engl) 132 (7):827-833. doi:10.1097/cm9.0000000000000153
62. Gao F, Zhang B, Hu B, Lu M, An M, Liu Y, Fang Y, Zhao G, Shi C, Zhou J, Liu Y, Li C (2020) Outcomes of Hip Arthroscopy for Femoroacetabular Impingement in Chinese Patients Aged 50 Years or Older. Orthop Surg 12 (3):843-851. doi:10.1111/os.12688
63. Gao G, Zhou C, Ao Y, Wang J, Xu Y (2022) Variations in postoperative electrolyte concentrations and influential factors in hip arthroscopy.

BMC Musculoskelet Disord 23 (1):473. doi:10.1186/s12891-022-05451-1
64. Gao G, Jiao C, Liu J, Zhou C, Liu Y, Ao Y, Xu Y (2022) Healing of joint capsule after hip arthroscopy using interportal capsulotomy and capsular closure influences clinical outcomes. J Orthop Surg Res 17 (1):316. doi:10.1186/s13018-022-03208-z
65. Gebhardt S, Hofer A, Wassilew GI, Sobau C, Zimmerer A (2023) Minced Cartilage Implantation in Acetabular Cartilage Defects: Case Series with 2-Year Results. Cartilage 14 (4):393-399. doi:10.1177/19476035231189840
66. Gédouin JE, Duperron D, Langlais F, Thomazeau H (2010) Update to femoroacetabular impingement arthroscopic management. Orthop Traumatol Surg Res 96 (3):222-227. doi:10.1016/j.otsr.2009.12.002
67. Gedouin JE, May O, Bonin N, Nogier A, Boyer T, Sadri H, Villar RN, Laude F (2010) Assessment of arthroscopic management of femoroacetabular impingement. A prospective multicenter study. Orthop Traumatol Surg Res 96 (8 Suppl):S59-67. doi:10.1016/j.otsr.2010.08.002
68. Gicquel T, Gédouin JE, Krantz N, May O, Gicquel P, Bonin N (2014) Function and osteoarthritis progression after arthroscopic treatment of femoro-acetabular impingement: a prospective study after a mean follow-up of 4.6 (4.2-5.5) years. Orthop Traumatol Surg Res 100 (6):651-656. doi:10.1016/j.otsr.2014.07.008
69. Giordano BD, Kuhns BD, Perets I, Yuen L, Domb BG (2020) Acetabular Morphologic Characteristics Predict Early Conversion to Arthroplasty After Isolated Hip Arthroscopy for Femoroacetabular Impingement. Am J Sports Med 48 (1):188-196. doi:10.1177/0363546519888894
70. Glaws KR, Ellis TJ, Hewett TE, Di Stasi S (2019) Return to Sport Rates in Physically Active Individuals 6 Months After Arthroscopy for Femoroacetabular Impingement Syndrome. J Sport Rehabil 28 (6):570-575. doi:10.1123/jsr.2017-0138
71. Gupta A, Redmond JM, Stake CE, Finch NA, Dunne KF, Domb BG (2014) Does the femoral cam lesion regrow after osteoplasty for femoroacetabular impingement? Two-year follow-up. Am J Sports Med 42 (9):2149-2155. doi:10.1177/0363546514541782
72. Gupta A, Redmond JM, Hammarstedt JE, Stake CE, Domb BG (2015) Does obesity affect outcomes in hip arthroscopy? A matched-pair controlled study with minimum 2-year follow-up. Am J Sports Med 43 (4):965-971. doi:10.1177/0363546514565089
73. Gürsan O, Hapa O, Matsuda DK, Aydemir S, Çeltik M, Cici H, Acan AE (2023) Postoperative alpha angle seems to be important for the achievement of clinical significance at a minimum 5-year follow-up after primary hip arthroscopy. J Hip Preserv Surg 10 (2):123-128. doi:10.1093/jhps/hnad010
74. Ha YC, Lim JY, Won YS, Lee YK, Koo KH, Kim JW (2020) Outcomes of arthroscopic femoroplasty in patients with cam lesions: Minimum 2-year follow-up. J Orthop Surg (Hong Kong) 28 (2):2309499020942049. doi:10.1177/2309499020942049
75. Haefeli PC, Albers CE, Steppacher SD, Tannast M, Büchler L (2017) What Are the Risk Factors for Revision Surgery After Hip Arthroscopy for Femoroacetabular Impingement at 7-year Followup? Clin Orthop Relat Res 475 (4):1169-1177. doi:10.1007/s11999-016-5115-6
76. Hartigan DE, Perets I, Yuen LC, Domb BG (2017) Results of hip arthroscopy in patients with MRI diagnosis of subchondral cysts-a case series. J Hip Preserv Surg 4 (4):324-331. doi:10.1093/jhps/hnx034
77. Hartmann A, Günther KP (2009) Arthroscopically assisted anterior decompression for femoroacetabular impingement: technique and early clinical results. Arch Orthop Trauma Surg 129 (8):1001-1009. doi:10.1007/s00402-008-0806-4
78. Hartwell MJ, Morgan AM, Nelson PA, Fernandez CE, Nicolay RW, Sheth U, Tjong VK, Terry MA (2021) Isolated Acetabuloplasty for Femoroacetabular Impingement: Favorable Patient-Reported Outcomes and Sustained Survivorship at Minimum 5-Year Follow-Up. Arthroscopy 37 (11):3288-3294. doi:10.1016/j.arthro.2021.03.080
79. Haskel JD, Baron SL, Zusmanovich M, Youm T (2020) Does Concomitant Lumbar Spine Disease Adversely Affect the Outcomes of Patients Undergoing Hip Arthroscopy? Am J Sports Med 48 (9):2178-2184. doi:10.1177/0363546520929344
80. Hassebrock JD, Krych AJ, Domb BG, Levy BA, Neville MR, Hartigan DE (2019) Bilateral Hip Arthroscopy: Can Results From Initial Arthroscopy for Femoroacetabular Impingement Predict Future Contralateral Results? Arthroscopy 35 (6):1837-1844.

doi:10.1016/j.arthro.2018.12.033
81. Hassebrock JD, Chhabra A, Makovicka JL, Economopoulos KJ (2020) Bilateral Hip Arthroscopy in High-Level Athletes: Results of a Shorter Interval Between Staged Bilateral Hip Arthroscopies. Am J Sports Med 48 (3):654-660. doi:10.1177/0363546519895259
82. Hassebrock JD, Makovicka JL, Chhabra A, Anastasi MB, Menzer HM, Wilcox JG, Economopoulos KJ (2020) Hip Arthroscopy in the High-Level Athlete: Does Capsular Closure Make a Difference? Am J Sports Med 48 (10):2465-2470. doi:10.1177/0363546520936255
83. Haviv B, Singh PJ, Takla A, O'Donnell J (2010) Arthroscopic femoral osteochondroplasty for cam lesions with isolated acetabular chondral damage. J Bone Joint Surg Br 92 (5):629-633. doi:10.1302/0301-620x.92b5.23667
84. Haviv B, O'Donnell J (2010) Arthroscopic treatment for symptomatic bilateral cam-type femoroacetabular impingement. Orthopedics 33 (12):874. doi:10.3928/01477447-20101021-14
85. Hevesi M, Hartigan DE, Wu IT, Levy BA, Domb BG, Krych AJ (2018) Are Results of Arthroscopic Labral Repair Durable in Dysplasia at Midterm Follow-up? A 2-Center Matched Cohort Analysis. Am J Sports Med 46 (7):1674-1684. doi:10.1177/0363546518767399
86. Holleyman R, Sohatee MA, Lyman S, Malviya A, Khanduja V (2023) Hip arthroscopy for femoroacetabular impingement is associated with significant improvement in early patient reported outcomes: analysis of 4963 cases from the UK non-arthroplasty registry (NAHR) dataset. Knee Surg Sports Traumatol Arthrosc 31 (1):58-69. doi:10.1007/s00167-022-07042-y
87. Honda E, Utsunomiya H, Hatakeyama A, Nakashima H, Suzuki H, Matsuda DK, Sakai A, Uchida S (2020) Patients aged in their 70s do not have a high risk of progressive osteoarthritis following arthroscopic femoroacetabular impingement correction and labral preservation surgery. Knee Surg Sports Traumatol Arthrosc 28 (5):1648-1655. doi:10.1007/s00167-019-05520-4
88. Horisberger M, Brunner A, Herzog RF (2010) Arthroscopic treatment of femoral acetabular impingement in patients with preoperative generalized degenerative changes. Arthroscopy 26 (5):623-629. doi:10.1016/j.arthro.2009.09.003
89. Horisberger M, Brunner A, Herzog RF (2010) Arthroscopic treatment of femoroacetabular impingement of the hip: a new technique to access the joint. Clin Orthop Relat Res 468 (1):182-190. doi:10.1007/s11999-009-1005-5
90. Hufeland M, Krüger D, Haas NP, Perka C, Schröder JH (2016) Arthroscopic treatment of femoroacetabular impingement shows persistent clinical improvement in the mid-term. Arch Orthop Trauma Surg 136 (5):687-691. doi:10.1007/s00402-016-2427-7
91. Jimenez AE, Lee MS, Owens JS, Maldonado DR, Saks BR, Lall AC, Domb BG (2022) Effect of Cigarette Smoking on Midterm Outcomes After Arthroscopic Surgery for Femoroacetabular Impingement Syndrome: A Propensity-Matched Controlled Study With Minimum 5-Year Follow-up. Orthop J Sports Med 10 (5):23259671221090905. doi:10.1177/23259671221090905
92. Ju XD, He ZY, Dang HH, Zhang X, Zhang Z, Xu Y, Huang HJ, Wang JQ (2023) Relationship between the Depth of Acetabuloplasty and Outcomes of Hip Arthroscopy in Patients with Global Pincer Femoroacetabular Impingement: Study with a Minimum Follow-Up Period of 2 Years. Orthop Surg 15 (6):1571-1578. doi:10.1111/os.13739
93. Kaplan DJ, Matache BA, Fried J, Burke C, Samim M, Youm T (2021) Improved Functional Outcome Scores Associated with Greater Reduction in Cam Height Using the Femoroacetabular Impingement Resection Arc During Hip Arthroscopy. Arthroscopy 37 (12):3455-3465. doi:10.1016/j.arthro.2021.05.014
94. Knapik DM, Clapp IM, Wichman D, Nho SJ (2021) Use of Younger Patient Age and Greater Anterior Center-Edge Angle to Predict the Need for Bilateral Hip Arthroscopy in Patients With Bilateral Femoroacetabular Impingement-Related Hip Pain. Am J Sports Med 49 (8):2110-2116. doi:10.1177/03635465211015431
95. Kollmorgen R (2024) Editorial Commentary: The Addition of the Pericapsular Nerve Group Block Leads to Reduced Pain Up to 24 Hours After Hip Arthroscopy. Arthroscopy 40 (2):381-383. doi:10.1016/j.arthro.2023.07.014
96. Krych AJ, Thompson M, Knutson Z, Scoon J, Coleman SH (2013) Arthroscopic labral repair versus selective labral debridement in female

patients with femoroacetabular impingement: a prospective randomized study. Arthroscopy 29 (1):46-53. doi:10.1016/j.arthro.2012.07.011
97. Kunze KN, Beck EC, Nwachukwu BU, Ahn J, Nho SJ (2019) Early Hip Arthroscopy for Femoroacetabular Impingement Syndrome Provides Superior Outcomes When Compared With Delaying Surgical Treatment Beyond 6 Months. Am J Sports Med 47 (9):2038-2044. doi:10.1177/0363546519837192
98. Kunze KN, Beck EC, Okoroha KR, Chahla J, Suppauksorn S, Bush-Joseph CA, Katakam A, Nho SJ (2019) Effect of prior ipsilateral lower extremity surgery on 2-year outcomes following hip arthroscopy for femoroacetabular impingement syndrome. J Hip Preserv Surg 6 (3):241-248. doi:10.1093/jhps/hnz031
99. Kunze KN, Nwachukwu BU, Beck EC, Chahla J, Gowd AK, Rasio J, Nho SJ (2020) Preoperative Duration of Symptoms Is Associated With Outcomes 5 Years After Hip Arthroscopy for Femoroacetabular Impingement Syndrome. Arthroscopy 36 (4):1022-1029. doi:10.1016/j.arthro.2019.08.032
100. LaFrance R, Kenney R, Giordano B, Mohr K, Cabrera J, Snibbe J (2015) The effect of platelet enriched plasma on clinical outcomes in patients with femoroacetabular impingement following arthroscopic labral repair and femoral neck osteoplasty. J Hip Preserv Surg 2 (2):158-163. doi:10.1093/jhps/hnv023
101. Lall AC, Battaglia MR, Maldonado DR, Perets I, Laseter JR, Go CC, Domb BG (2019) Does Femoral Retroversion Adversely Affect Outcomes After Hip Arthroscopy for Femoroacetabular Impingement Syndrome? A Midterm Analysis. Arthroscopy 35 (11):3035-3046. doi:10.1016/j.arthro.2019.03.046
102. Larson CM, Giveans MR, Stone RM (2012) Arthroscopic debridement versus refixation of the acetabular labrum associated with femoroacetabular impingement: mean 3.5-year follow-up. Am J Sports Med 40 (5):1015-1021. doi:10.1177/0363546511434578
103. Larson CM, McGaver RS, Collette NR, Giveans MR, Ross JR, Bedi A, Nepple JJ (2019) Arthroscopic Surgery for Femoroacetabular Impingement in Skeletally Immature Athletes: Radiographic and Clinical Analysis. Arthroscopy 35 (6):1819-1825. doi:10.1016/j.arthro.2019.01.029
104. Larson CM, Giveans MR (2008) Arthroscopic management of femoroacetabular impingement: early outcomes measures. Arthroscopy 24 (5):540-546. doi:10.1016/j.arthro.2007.11.007
105. Larson CM, Giveans MR (2009) Arthroscopic debridement versus refixation of the acetabular labrum associated with femoroacetabular impingement. Arthroscopy 25 (4):369-376. doi:10.1016/j.arthro.2008.12.014
106. Laurito GM, Aranha FL, Piedade SR (2021) FUNCTIONAL OUTCOMES OF ARTHROSCOPIC TREATMENT IN 230 FEMOROACETABULAR IMPINGEMENT CASES. Acta Ortop Bras 29 (2):67-71. doi:10.1590/1413-785220212902236846
107. Lee JW, Hwang DS, Kang C, Hwang JM, Chung HJ (2019) Arthroscopic Repair of Acetabular Labral Tears Associated with Femoroacetabular Impingement: 7-10 Years of Long-Term Follow-up Results. Clin Orthop Surg 11 (1):28-35. doi:10.4055/cios.2019.11.1.28
108. Lee JK, Hwang DS, Kim SB, Kang C, Hwang JM, Lee GS, Park EJ (2021) The role and clinical relevance of the ligamentum teres: long-term outcomes after hip arthroscopic surgery of cam-type femoroacetabular impingement. J Hip Preserv Surg 8 (4):360-366. doi:10.1093/jhps/hnab080
109. Lee MS, Jimenez AE, Owens JS, Curley AJ, Paraschos OA, Maldonado DR, Lall AC, Domb BG (2022) Comparison of Outcomes Between Nonsmokers and Patients Who Discontinued Smoking 1 Month Before Primary Hip Arthroscopy: A Propensity-Matched Study With Minimum 2-Year Follow-up. Orthop J Sports Med 10 (6):23259671221097372. doi:10.1177/23259671221097372
110. Lee SM, Kim JS, Moon NH, Woo SH, Park C, Shin WC (2023) Recovery After Hip Arthroscopy in Patients With Combined Femoroacetabular Impingement and Labral Tears Compared With Isolated Pathology. Orthop J Sports Med 11 (6):23259671231167908. doi:10.1177/23259671231167908
111. Levy DM, Kuhns BD, Frank RM, Grzybowski JS, Campbell KA, Brown S, Nho SJ (2017) High Rate of Return to Running for Athletes After

Hip Arthroscopy for the Treatment of Femoroacetabular Impingement and Capsular Plication. Am J Sports Med 45 (1):127-134. doi:10.1177/0363546516664883
112. Levy DM, Cvetanovich GL, Kuhns BD, Greenberg MJ, Alter JM, Nho SJ (2017) Hip Arthroscopy for Atypical Posterior Hip Pain: A Comparative Matched-Pair Analysis. Am J Sports Med 45 (7):1627-1632. doi:10.1177/0363546517692983
113. Lin LJ, Akpinar B, Bloom DA, Youm T (2021) Age and Outcomes in Hip Arthroscopy for Femoroacetabular Impingement: A Comparison Across 3 Age Groups. Am J Sports Med 49 (1):82-89. doi:10.1177/0363546520974370
114. Lin CC, Colasanti CA, Bloom DA, Youm T (2021) Six-Month Outcome Scores Predicts Short-Term Outcomes After Hip Arthroscopy. Arthroscopy 37 (10):3081-3087. doi:10.1016/j.arthro.2021.03.046
115. Lindman I, Öhlin A, Desai N, Samuelsson K, Ayeni OR, Hamrin Senorski E, Sansone M (2020) Five-Year Outcomes After Arthroscopic Surgery for Femoroacetabular Impingement Syndrome in Elite Athletes. Am J Sports Med 48 (6):1416-1422. doi:10.1177/0363546520908840
116. Lindman I, Abrahamsson J, Öhlin A, Wörner T, Eek F, Ayeni OR, Senorski EH, Sansone M (2021) Improvements After Arthroscopic Treatment for Femoroacetabular Impingement Syndrome in High-Level Ice Hockey Players: 2-Year Outcomes by Player Position. Orthop J Sports Med 9 (3):2325967120981687. doi:10.1177/2325967120981687
117. Litrenta J, Mu B, Chen AW, Ortiz-Declet V, Perets I, Domb BG (2019) Radiographic and Clinical Outcomes of Adolescents With Acetabular Retroversion Treated Arthroscopically. J Pediatr Orthop 39 (10):510-515. doi:10.1097/bpo.0000000000001063
118. Litrenta J, Mu BH, Ortiz-Declet V, Chen AW, Perets I, Wojnowski NM, Domb BG (2020) Hip Arthroscopy Successfully Treats Femoroacetabular Impingement in Adolescent Athletes. J Pediatr Orthop 40 (3):e156-e160. doi:10.1097/bpo.0000000000001411
119. Maimaitimin M, Yang F, Huang HJ, Xu Y, Zhang X, Ao YF, Wang JQ (2022) Outcomes After Hip Arthroscopy for Femoroacetabular Impingement Syndrome in Chinese Patients: A Minimum 2-Year Follow-up Study. Orthop J Sports Med 10 (11):23259671221135218. doi:10.1177/23259671221135218
120. Maldonado DR, Krych AJ, Levy BA, Hartigan DE, Laseter JR, Domb BG (2018) Does Iliopsoas Lengthening Adversely Affect Clinical Outcomes After Hip Arthroscopy? A Multicenter Comparative Study. Am J Sports Med 46 (11):2624-2631. doi:10.1177/0363546518785966
121. Maldonado DR, Laseter JR, Perets I, Ortiz-Declet V, Chen AW, Lall AC, Domb BG (2019) The Effect of Complete Tearing of the Ligamentum Teres in Patients Undergoing Primary Hip Arthroscopy for Femoroacetabular Impingement and Labral Tears: A Match-Controlled Study. Arthroscopy 35 (1):80-88. doi:10.1016/j.arthro.2018.07.042
122. Maldonado DR, Go CC, Laseter JR, Lall AC, Kopscik MR, Domb BG (2019) Primary labral reconstruction in patients with femoroacetabular impingement, irreparable labral tears and severe acetabular chondral defects decreases the risk of conversion to total hip arthroplasty: a pair-matched study. J Hip Preserv Surg 6 (3):214-226. doi:10.1093/jhps/hnz028
123. Maldonado DR, Diulus SC, Shapira J, Rosinsky PJ, Kyin C, Ankem HK, Lall AC, Domb BG (2021) Hip Arthroscopic Surgery in the Context of Femoroacetabular Impingement Syndrome, Labral Tear, and Acetabular Overcoverage: Minimum 5-Year Outcomes With a Subanalysis Against Patients Without Overcoverage. Am J Sports Med 49 (1):55-65. doi:10.1177/0363546520969985
124. Maldonado DR, Yelton MJ, Rosinsky PJ, Shapira J, Meghpara MB, Lall AC, Domb BG (2020) Return to play after hip arthroscopy among tennis players: outcomes with minimum five-year follow-up. BMC Musculoskelet Disord 21 (1):400. doi:10.1186/s12891-020-03424-w
125. Mardones R, Via AG, Tomic A, Rodriguez C, Salineros M, Somarriva M (2016) Arthroscopic release of iliopsoas tendon in patients with femoro-acetabular impingement: clinical results at mid-term follow-up. Muscles Ligaments Tendons J 6 (3):378-383. doi:10.11138/mltj/2016.6.3.378
126. Marom N, Olsen R, Burger JA, Dooley MS, Coleman SH, Ranawat AS, Kelly BT, Nawabi DH (2023) Majority of competitive soccer players return to soccer following hip arthroscopy for femoroacetabular impingement: female and older aged players are less likely to return to soccer. Knee

Surg Sports Traumatol Arthrosc 31 (7):2721-2729. doi:10.1007/s00167-023-07349-4
127. Ippolito JA, Martinez M, Thomson JE, Willis AR, Beebe KS, Patterson FR, Benevenia J (2019) Complications following allograft reconstruction for primary bone tumors: Considerations for management. J Orthop 16 (1):49-54. doi:10.1016/j.jor.2018.12.013
128. Mas Martinez J, Sanz-Reig J, Verdu Roman C, Bustamante Suarez de Puga D, Martinez Gimenez E, Morales Santias M (2020) Recreational Sports and Intra-articular Hip Injuries in Patients Undergoing Hip Arthroscopy for Femoroacetabular Impingement. Arthrosc Sports Med Rehabil 2 (4):e321-e328. doi:10.1016/j.asmr.2020.04.005
129. Más Martínez J, Cuenca Copete A, Verdú Román C, Jiménez Arias D, Beneito Pastor D, Sanz-Reig J (2024) Hip arthroscopy for femoroacetabular impingement with 10-year minimum follow-up. Rev Esp Cir Ortop Traumatol 68 (1):35-43. doi:10.1016/j.recot.2023.06.015
130. Martinot P, Trouillez T, Dartus J, Putman S, Girard J, Migaud H (2020) Treatment of femoroacetabular impingement by arthroscopy versus anterior mini-open approach: Case-control study of a continuous series of 91 cases at a mean 4.6 years' follow-up. Orthop Traumatol Surg Res 106 (8):1575-1580. doi:10.1016/j.otsr.2020.07.006
131. Matsuda DK, Gupta N, Burchette RJ, Sehgal B (2015) Arthroscopic surgery for global versus focal pincer femoroacetabular impingement: are the outcomes different? J Hip Preserv Surg 2 (1):42-50. doi:10.1093/jhps/hnv010
132. Matsuda D, Kivlan BR, Nho SJ, Wolff AB, Salvo JP, Jr., Christoforetti JJ, Martin RL, Carreira DS (2021) Tenotomy for Iliopsoas Pathology is Infrequently Performed and Associated with Poorer Outcomes in Hips Undergoing Arthroscopy for Femoroacetabular Impingement. Arthroscopy 37 (7):2140-2148. doi:10.1016/j.arthro.2021.02.018
133. May O, Ouattara K, Flecher X, Wettstein M (2020) Does labral repair have a clinical benefit during arthroscopic treatment of femoro-acetabular impingement? Prospective multicentre study with 2-year follow-up. Orthop Traumatol Surg Res 106 (8s):S237-s241. doi:10.1016/j.otsr.2020.09.002
134. McConkey MO, Chadayammuri V, Garabekyan T, Mayer SW, Kraeutler MJ, Mei-Dan O (2019) Simultaneous Bilateral Hip Arthroscopy in Adolescent Athletes With Symptomatic Femoroacetabular Impingement. J Pediatr Orthop 39 (4):193-197. doi:10.1097/bpo.0000000000000987
135. Menge TJ, Briggs KK, Rahl MD, Philippon MJ (2021) Hip Arthroscopy for Femoroacetabular Impingement in Adolescents: 10-Year Patient-Reported Outcomes. Am J Sports Med 49 (1):76-81. doi:10.1177/0363546520973977
136. Mercier M, Dangin A, Ollier E, Bonin N (2019) Does acetabular dysplasia affect outcome in arthroscopic treatment of cam femoroacetabular impingement? Case-control study with and without acetabular dysplasia. Orthop Traumatol Surg Res 105 (1):7-10. doi:10.1016/j.otsr.2018.10.011
137. Migliorini F, Baroncini A, Eschweiler J, Knobe M, Tingart M, Maffulli N (2023) Return to sport after arthroscopic surgery for femoroacetabular impingement. Surgeon 21 (1):21-30. doi:10.1016/j.surge.2021.11.006
138. Mihalič R, Brumat P, Trebše R (2021) A novel concept of cam-type femoroacetabular impingement treatment with patient-specific template guided osteochondral deformity ablation. Int Orthop 45 (4):907-913. doi:10.1007/s00264-020-04887-1
139. Mohan R, Johnson NR, Hevesi M, Gibbs CM, Levy BA, Krych AJ (2017) Return to Sport and Clinical Outcomes After Hip Arthroscopic Labral Repair in Young Amateur Athletes: Minimum 2-Year Follow-Up. Arthroscopy 33 (9):1679-1684. doi:10.1016/j.arthro.2017.03.011
140. Moon JK, Yoon JY, Kim CH, Lee S, Kekatpure AL, Yoon PW (2020) Hip Arthroscopy for Femoroacetabular Impingement and Concomitant Labral Tears: A Minimum 2-Year Follow-Up Study. Arthroscopy 36 (8):2186-2194. doi:10.1016/j.arthro.2020.04.041
141. Moriya M, Fukushima K, Uchiyama K, Takahira N, Yamamoto T, Minegishi Y, Takaso M (2017) Clinical results of arthroscopic surgery in patients over 50 years of age-what viability does it have as a joint preservative surgery? J Orthop Surg Res 12 (1):2. doi:10.1186/s13018-016-0504-9
142. Mortensen AJ, Metz AK, Featherall J, O'Neill DC, Rosenthal RM, Aoki SK (2023) Hip Joint Venting Decreases the Traction Force Required to Access the Central Compartment During Hip Arthroscopy. Arthrosc Sports Med Rehabil 5 (3):e589-e596. doi:10.1016/j.asmr.2023.02.011

143. Mullins K, Hanlon M, Carton P (2020) Arthroscopic correction of femoroacetabular impingement improves athletic performance in male athletes. Knee Surg Sports Traumatol Arthrosc 28 (7):2285-2294. doi:10.1007/s00167-019-05683-0
144. Mullins K, Filan D, Carton P (2021) Arthroscopic Correction of Sports-Related Femoroacetabular Impingement in Competitive Athletes: 2-Year Clinical Outcome and Predictors for Achieving Minimal Clinically Important Difference. Orthop J Sports Med 9 (3):2325967121989675. doi:10.1177/2325967121989675
145. Nakashima H, Tsukamoto M, Ohnishi Y, Utsunomiya H, Kanezaki S, Sakai A, Uchida S (2019) Clinical and Radiographic Predictors for Unsalvageable Labral Tear at the Time of Initial Hip Arthroscopic Management for Femoroacetabular Impingement. Am J Sports Med 47 (9):2029-2037. doi:10.1177/0363546519856018
146. Nakashima H, Utsunomiya H, Kanezaki S, Suzuki H, Nakamura E, Larson CM, Sakai A, Uchida S (2021) Is Arthroscopic Hip Labral Repair/Reconstruction Surgery Effective for Treating Femoroacetabular Impingement in the Presence of Osteoarthritis? Clin J Sport Med 31 (4):367-373. doi:10.1097/jsm.0000000000000768
147. Nawabi DH, Degen RM, Fields KG, McLawhorn A, Ranawat AS, Sink EL, Kelly BT (2016) Outcomes After Arthroscopic Treatment of Femoroacetabular Impingement for Patients With Borderline Hip Dysplasia. Am J Sports Med 44 (4):1017-1023. doi:10.1177/0363546515624682
148. Nguyen TQ, Friedman JM, Flores SE, Zhang AL (2020) Fast Starters and Slow Starters After Hip Arthroscopy for Femoroacetabular Impingement: Correlation of Early Postoperative Pain and 2-Year Outcomes. Am J Sports Med 48 (12):2903-2909. doi:10.1177/0363546520952406
149. Nho SJ, Magennis EM, Singh CK, Kelly BT (2011) Outcomes after the arthroscopic treatment of femoroacetabular impingement in a mixed group of high-level athletes. Am J Sports Med 39 Suppl:14s-19s. doi:10.1177/0363546511401900
150. Nielsen TG, Miller LL, Lund B, Christiansen SE, Lind M (2014) Outcome of arthroscopic treatment for symptomatic femoroacetabular impingement. BMC Musculoskelet Disord 15:394. doi:10.1186/1471-2474-15-394
151. Nwachukwu BU, Beck EC, Kunze KN, Chahla J, Rasio J, Nho SJ (2020) Defining the Clinically Meaningful Outcomes for Arthroscopic Treatment of Femoroacetabular Impingement Syndrome at Minimum 5-Year Follow-up. Am J Sports Med 48 (4):901-907. doi:10.1177/0363546520902736
152. Öhlin A, Ahldén M, Lindman I, Jónasson P, Desai N, Baranto A, Ayeni OR, Sansone M (2020) Good 5-year outcomes after arthroscopic treatment for femoroacetabular impingement syndrome. Knee Surg Sports Traumatol Arthrosc 28 (4):1311-1316. doi:10.1007/s00167-019-05429-y
153. Ohlsen SM, Metz AK, Froerer DL, Mortensen AJ, Smith TR, Featherall J, Rosenthal RM, Aoki SK (2024) Relationship Between Hip Capsular Thickness and Instability After Previous Hip Arthroscopic Surgery: A Matched-Cohort Analysis. Orthop J Sports Med 12 (3):23259671241231763. doi:10.1177/23259671241231763
154. Olivero M, Capurro B, Reis-Campos P, Aprato A, Ayeni O, Chawla A, Larrainzar Garijo R, Marín-Peña O (2023) Low-dose prophylaxis protocol for heterotopic ossification after hip preservation surgery in a sport participants cohort. Sicot j 9:27. doi:10.1051/sicotj/2023024
155. O'Reilly OC, Day MA, Seiffert K, Fritts HM, An Q, Westermann RW, Larson CM (2022) Male Gender and Competitive Athlete Status Are Associated With Better Outcomes Following Hip Arthroscopy In Patients With Global Acetabular Retroversion. Arthrosc Sports Med Rehabil 4 (5):e1721-e1729. doi:10.1016/j.asmr.2022.06.019
156. Ortiz-Declet V, Yuen LC, Schwarzman GR, Chen AW, Perets I, Domb BG (2020) Return to Play in Amateur Soccer Players Undergoing Hip Arthroscopy: Short- to Mid-Term Follow-Up. Arthroscopy 36 (2):442-449. doi:10.1016/j.arthro.2019.08.027
157. Owens JS, Jimenez AE, Lee MS, Maldonado DR, Lall AC, Domb BG (2022) Outcomes and Return-to-Sport Rates for Elite Athletes With Femoral Retroversion Undergoing Hip Arthroscopy: A Propensity-Matched Analysis With Minimum 2-Year Follow-up. Orthop J Sports Med 10 (6):23259671221099840. doi:10.1177/23259671221099840

158. Owens JS, Lee MS, Jimenez AE, Maldonado DR, Paraschos OA, Domb BG (2022) Sex-Based Differences in Athletes Undergoing Primary Hip Arthroscopy With Labral Reconstruction: A Propensity-Matched Analysis With Minimum 2-Year Follow-up. Orthop J Sports Med 10 (6):23259671221100861. doi:10.1177/23259671221100861
159. Özbek EA, Ayduğan MY, Akmeşe R (2021) The effectiveness of peripheral compartment first access and periportal capsulotomy technique for arthroscopic management of femoroacetabular impingement: A prospective case series. Acta Orthop Traumatol Turc 55 (6):486-492. doi:10.5152/j.aott.2021.21174
160. Palmer DH, Ganesh V, Comfort T, Tatman P (2012) Midterm outcomes in patients with cam femoroacetabular impingement treated arthroscopically. Arthroscopy 28 (11):1671-1681. doi:10.1016/j.arthro.2012.04.154
161. Pansard E, Thaunat M, Vigan M, Wettstein M, Flecher X (2021) Impact of bone deformities and labral and cartilage lesions on early functional results of arthroscopic treatment of femoroacetabular impingement. Orthop Traumatol Surg Res 107 (8s):103069. doi:10.1016/j.otsr.2021.103069
162. Parvaresh K, Rasio JP, Wichman D, Chahla J, Nho SJ (2021) The Influence of Body Mass Index on Outcomes After Hip Arthroscopy for Femoroacetabular Impingement Syndrome: Five-Year Results in 140 Patients. Am J Sports Med 49 (1):90-96. doi:10.1177/0363546520976357
163. Parvaresh K, Rasio JP, Martin RL, Kivlan BR, Carreira D, Christoforetti JJ, Harris JD, Matsuda DK, Salvo J, Wolff AB, Nho SJ (2021) Achievement of Meaningful Clinical Outcomes Is Unaffected by Capsulotomy Type During Arthroscopic Treatment of Femoroacetabular Impingement Syndrome: Results From the Multicenter Arthroscopic Study of the Hip (MASH) Study Group. Am J Sports Med 49 (3):713-720. doi:10.1177/0363546520987226
164. Parvaresh KC, Wichman DM, Alter TD, Clapp IM, Nho SJ (2021) High rate of return to tennis after hip arthroscopy for patients with femoroacetabular impingement syndrome. Phys Ther Sport 51:45-49. doi:10.1016/j.ptsp.2021.06.007
165. Perets I, Gupta A, Chaharbakhshi EO, Ashberg L, Hartigan DE, Close MR, Domb BG (2017) Does Bony Regrowth Occur After Arthroscopic Femoroplasty in a Group of Young Adolescents? Arthroscopy 33 (5):988-995. doi:10.1016/j.arthro.2017.01.023
166. Perets I, Rybalko D, Chaharbakhshi EO, Mu BH, Chen AW, Domb BG (2018) Minimum Five-Year Outcomes of Hip Arthroscopy for the Treatment of Femoroacetabular Impingement and Labral Tears in Patients with Obesity: A Match-Controlled Study. J Bone Joint Surg Am 100 (11):965-973. doi:10.2106/jbjs.17.00892
167. Perets I, Chaharbakhshi EO, Mansor Y, Ashberg LJ, Mu BH, Battaglia MR, Lall AC, Domb BG (2019) Midterm Outcomes of Iliopsoas Fractional Lengthening for Internal Snapping as a Part of Hip Arthroscopy for Femoroacetabular Impingement and Labral Tear: A Matched Control Study. Arthroscopy 35 (5):1432-1440. doi:10.1016/j.arthro.2018.11.050
168. Perets I, Chaharbakhshi EO, Shapira J, Ashberg L, Mu BH, Domb BG (2019) Hip Arthroscopy for Femoroacetabular Impingement and Labral Tears in Patients Younger than 50 Years: Minimum Five-year Outcomes, Survivorship, and Risk Factors for Reoperations. J Am Acad Orthop Surg 27 (4):e173-e183. doi:10.5435/jaaos-d-17-00258
169. Philippon M, Schenker M, Briggs K, Kuppersmith D (2007) Femoroacetabular impingement in 45 professional athletes: associated pathologies and return to sport following arthroscopic decompression. Knee Surg Sports Traumatol Arthrosc 15 (7):908-914. doi:10.1007/s00167-007-0332-x
170. Philippon MJ, Briggs KK, Yen YM, Kuppersmith DA (2009) Outcomes following hip arthroscopy for femoroacetabular impingement with associated chondrolabral dysfunction: minimum two-year follow-up. J Bone Joint Surg Br 91 (1):16-23. doi:10.1302/0301-620x.91b1.21329
171. Philippon MJ, Weiss DR, Kuppersmith DA, Briggs KK, Hay CJ (2010) Arthroscopic labral repair and treatment of femoroacetabular impingement in professional hockey players. Am J Sports Med 38 (1):99-104. doi:10.1177/0363546509346393
172. Philippon MJ, Schroder ESBG, Briggs KK (2012) Hip arthroscopy for femoroacetabular impingement in patients aged 50 years or older. Arthroscopy 28 (1):59-65. doi:10.1016/j.arthro.2011.07.004
173. Philippon MJ, Ejnisman L, Ellis HB, Briggs KK (2012) Outcomes 2 to 5 years following hip arthroscopy for femoroacetabular impingement in

the patient aged 11 to 16 years. Arthroscopy 28 (9):1255-1261. doi:10.1016/j.arthro.2012.02.006
174. Polesello GC, Queiroz MC, Ono NK, Honda EK, Guimarães RP, Junior WR (2009) ARTHROSCOPIC TREATMENT OF FEMOROACETABULAR IMPINGEMENT. Rev Bras Ortop 44 (3):230-238. doi:10.1016/s2255-4971(15)30073-2
175. Polesello GC, Lima FR, Guimaraes RP, Ricioli W, Queiroz MC (2014) Arthroscopic treatment of femoroacetabular impingement: minimum five-year follow-up. Hip Int 24 (4):381-386. doi:10.5301/hipint.5000135
176. Pontiff M, Ithurburn MP, Ellis T, Cenkus K, Stasi SD (2016) PRE- AND POST-OPERATIVE SELF-REPORTED FUNCTION AND QUALITY OF LIFE IN WOMEN WITH AND WITHOUT GENERALIZED JOINT LAXITY UNDERGOING HIP ARTHROSCOPY FOR FEMOROACETABULAR IMPINGEMENT. Int J Sports Phys Ther 11 (3):378-387
177. Rafols C, Monckeberg JE, Numair J, Botello J, Rosales J (2015) Platelet-Rich Plasma Augmentation of Arthroscopic Hip Surgery for Femoroacetabular Impingement: A Prospective Study With 24-Month Follow-up. Arthroscopy 31 (10):1886-1892. doi:10.1016/j.arthro.2015.03.025
178. Ramos N, Youssefzadeh K, Gerhardt M, Banffy M (2020) Results of hip arthroscopy in elite level water polo players with femoroacetabular impingement: return to play and patient satisfaction. J Hip Preserv Surg 7 (1):116-121. doi:10.1093/jhps/hnz069
179. Rhee SM, Kang SY, Jang EC, Kim JY, Ha YC (2016) Clinical outcomes after arthroscopic acetabular labral repair using knot-tying or knotless suture technique. Arch Orthop Trauma Surg 136 (10):1411-1416. doi:10.1007/s00402-016-2505-x
180. Riedl M, Banke IJ, Goronzy J, Sobau C, Steimer O, Thier S, Zinser W, Henssler L, Alt V, Fickert S (2022) Patients with Small Acetabular Cartilage Defects Caused by Femoroacetabular Impingement Do Not Benefit from Microfracture. J Clin Med 11 (21). doi:10.3390/jcm11216283
181. Rivera E, Seijas R, Rubio M, García-Balletbó M, Vilar JM, Boada PL, Cugat R (2020) Outcomes at 2-Years Follow-Up After Hip Arthroscopy Combining Bone Marrow Concentrate. J Invest Surg 33 (7):655-663. doi:10.1080/08941939.2018.1535010
182. Rosinsky PJ, Kyin C, Lall AC, Shapira J, Maldonado DR, Domb BG (2019) Rate of Return to Sport and Functional Outcomes After Bilateral Hip Arthroscopy in High-Level Athletes. Am J Sports Med 47 (14):3444-3454. doi:10.1177/0363546519885354
183. Said HG, Masoud MA, Morsi MMA, El-Assal MA (2019) Outcomes of hip arthroscopy for femoroacetabular impingement: The effect of morphological type and chondrolabral damage. Sicot j 5:16. doi:10.1051/sicotj/2019012
184. Saito M, Utsunomiya H, Hatakeyama A, Nakashima H, Nishimura H, Matsuda DK, Sakai A, Uchida S (2019) Hip Arthroscopic Management Can Improve Osteitis Pubis and Bone Marrow Edema in Competitive Soccer Players With Femoroacetabular Impingement. Am J Sports Med 47 (2):408-419. doi:10.1177/0363546518819099
185. Saks BR, Glein RM, Jimenez AE, Ankem HK, Sabetian PW, Maldonado DR, Lall AC, Domb BG (2022) Patients Obtain Meaningful Clinical Benefit After Hip Arthroscopy Despite Preoperative Psychological Distress: A Propensity-Matched Analysis of Mid-Term Outcomes. Arthroscopy 38 (3):773-782. doi:10.1016/j.arthro.2021.05.010
186. Sanders TL, Reardon P, Levy BA, Krych AJ (2017) Arthroscopic treatment of global pincer-type femoroacetabular impingement. Knee Surg Sports Traumatol Arthrosc 25 (1):31-35. doi:10.1007/s00167-016-4266-z
187. Sansone M, Ahldén M, Jonasson P, Thomeé C, Swärd L, Collin D, Baranto A, Karlsson J, Thomeé R (2016) Outcome of hip arthroscopy in patients with mild to moderate osteoarthritis-A prospective study. J Hip Preserv Surg 3 (1):61-67. doi:10.1093/jhps/hnv079
188. Sansone M, Ahldén M, Jonasson P, Thomeé C, Swärd L, Baranto A, Karlsson J, Thomeé R (2015) Good Results After Hip Arthroscopy for Femoroacetabular Impingement in Top-Level Athletes. Orthop J Sports Med 3 (2):2325967115569691. doi:10.1177/2325967115569691
189. Sansone M, Ahldén M, Jónasson P, Thomeé C, Swärd L, Öhlin A, Baranto A, Karlsson J, Thomeé R (2017) Outcome after hip arthroscopy for femoroacetabular impingement in 289 patients with minimum 2-year follow-up. Scand J Med Sci Sports 27 (2):230-235. doi:10.1111/sms.12641
190. Scanaliato JP, Chasteen J, Polmear MM, Salfiti C, Wolff AB (2020) Primary and Revision Circumferential Labral Reconstruction for Femoroacetabular Impingement in Athletes: Return to Sport and Technique. Arthroscopy 36 (10):2598-2610. doi:10.1016/j.arthro.2020.04.045

191. Schilders E, Dimitrakopoulou A, Bismil Q, Marchant P, Cooke C (2011) Arthroscopic treatment of labral tears in femoroacetabular impingement: a comparative study of refixation and resection with a minimum two-year follow-up. J Bone Joint Surg Br 93 (8):1027-1032. doi:10.1302/0301-620x.93b8.26065
192. Seijas R, Barastegui D, Celis CL, Montaña F, Cuscó X, Cugat R (2023) Heterotopic Ossification in Hip Arthroscopy. Surg J (N Y) 9 (1):e8-e12. doi:10.1055/s-0042-1758160
193. Shao JY, He ZY, Xu Y, Dai LH, Wang JQ, Ju XD (2023) Outcomes in Patients with Global Pincer Versus Focal Pincer Femoroacetabular Impingement Treated with Hip Arthroscopy: A Retrospective Study with a Minimum 2-Year Follow-Up. Orthop Surg 15 (1):223-229. doi:10.1111/os.13592
194. Shuang YJ, Mao Y, Yu KK, Li CB, Zhang MB (2024) Correlation Between Improvement in Pain After Ultrasound-Guided Intra-articular Hip Injection and Outcomes After Arthroscopy in Patients With Femoroacetabular Impingement. Orthop J Sports Med 12 (2):23259671231224497. doi:10.1177/23259671231224497
195. Singh PJ, O'Donnell JM (2010) The outcome of hip arthroscopy in Australian football league players: a review of 27 hips. Arthroscopy 26 (6):743-749. doi:10.1016/j.arthro.2009.10.010
196. Skendzel JG, Philippon MJ, Briggs KK, Goljan P (2014) The effect of joint space on midterm outcomes after arthroscopic hip surgery for femoroacetabular impingement. Am J Sports Med 42 (5):1127-1133. doi:10.1177/0363546514526357
197. Skowronek P, Synder M, Polguj M, Marczak D, Sibiński M (2017) Treatment of Femoroacetabular Impingement with a Mini-open Direct Anterior Approach. Indian J Orthop 51 (6):677-680. doi:10.4103/ortho.IJOrtho_248_16
198. Snaebjörnsson T, Anari SS, Lindman I, Desai N, Stålman A, Ayeni OR, Öhlin A (2022) Most Elite Athletes Who Underwent Hip Arthroscopy for Femoroacetabular Impingement Syndrome Did Not Return to the Same Level of Sport, but the Majority Were Satisfied With the Outcome of Surgery. Arthrosc Sports Med Rehabil 4 (3):e899-e906. doi:10.1016/j.asmr.2021.12.021
199. Sobti AS, Baryeh KW, Woolf R, Chana R (2020) Autologous matrix-induced chondrogenesis and bone marrow aspirate concentrate compared with microfracture for arthroscopic treatment of femoroacetabular impingement and chondral lesions of the hip: bridging the osteoarthritis gap and facilitating enhanced recovery. J Hip Preserv Surg 7 (3):503-510. doi:10.1093/jhps/hnaa047
200. Soriano KKJ, Flores SE, Aung MS, Nguyen TQ, Zhang AL (2021) Treatment of Labral Calcification in the Setting of Femoroacetabular Impingement Syndrome With Arthroscopic Calcification Excision, Labral Repair, and Osteoplasty Improves Outcomes. Arthroscopy 37 (2):554-563. doi:10.1016/j.arthro.2020.10.033
201. Stake CE, Jackson TJ, Stone JC, Domb BG (2013) Hip arthroscopy for labral tears in workers' compensation: a matched-pair controlled study. Am J Sports Med 41 (10):2302-2307. doi:10.1177/0363546513496055
202. Stone AV, Malloy P, Beck EC, Neal WH, Waterman BR, Bush-Joseph CA, Nho SJ (2019) Predictors of Persistent Postoperative Pain at Minimum 2 Years After Arthroscopic Treatment of Femoroacetabular Impingement. Am J Sports Med 47 (3):552-559. doi:10.1177/0363546518817538
203. Stone AV, Beck EC, Malloy P, Chahla J, Nwachukwu BU, Neal WH, Nho SJ (2019) Preoperative Predictors of Achieving Clinically Significant Athletic Functional Status After Hip Arthroscopy for Femoroacetabular Impingement at Minimum 2-Year Follow-Up. Arthroscopy 35 (11):3049-3056.e3041. doi:10.1016/j.arthro.2019.05.022
204. Stone AV, Mehta N, Beck EC, Waterman BR, Chahla J, Ukwuani G, Nho SJ (2019) Comparable patient-reported outcomes in females with or without joint hypermobility after hip arthroscopy and capsular plication for femoroacetabular impingement syndrome. J Hip Preserv Surg 6 (1):33-40. doi:10.1093/jhps/hnz004
205. Sugarman EP, Birns ME, Fishman M, Patel DN, Goldsmith L, Greene RS, Banffy MB (2021) Does Capsular Closure Affect Clinical

Outcomes in Hip Arthroscopy? A Prospective Randomized Controlled Trial. Orthop J Sports Med 9 (5):2325967120963110. doi:10.1177/2325967120963110
206. Sutton R, Yacovelli S, Vahedi H, Parvizi J (2021) Does a History of Slipped Capital Femoral Epiphysis in Patients Undergoing Femoroacetabular Osteoplasty for Femoroacetabular Impingement Affect Outcomes Scores or Risk of Reoperation? Clin Orthop Relat Res 479 (5):1028-1036. doi:10.1097/corr.0000000000001576
207. Thaunat M, Sarr S, Georgeokostas T, Azeem A, Murphy CG, Kacem S, Clowez G, Roberts T (2020) Femoroacetabular impingement treatment using the arthroscopic extracapsular outside-in approach: Does capsular suture affect functional outcome? Orthop Traumatol Surg Res 106 (3):569-575. doi:10.1016/j.otsr.2019.11.023
208. Tjong VK, Cogan CJ, Riederman BD, Terry MA (2016) A Qualitative Assessment of Return to Sport After Hip Arthroscopy for Femoroacetabular Impingement. Orthop J Sports Med 4 (11):2325967116671940. doi:10.1177/2325967116671940
209. Torabian KA, Cherian NJ, Dean MC, Eberlin CT, Kucharik MP, Dowley KS, LaPorte ZL, Martin SD (2023) Outcomes of Hip Arthroscopy in the Setting of Concomitant Symptomatic Lumbosacral Spine Pathology: A Matched Control Study With Minimum 24-Month Follow-up. Am J Sports Med 51 (12):3268-3279. doi:10.1177/03635465231197374
210. Torabian KA, Cherian NJ, Eberlin CT, Dean MC, Dowley KS, LaPorte ZL, Kucharik MP, Gillinov SM, Martin SD (2024) The Effect of Pelvic Incidence on Outcomes After Hip Arthroscopy for Femoroacetabular Impingement and Acetabular Labral Tears. Am J Sports Med 52 (3):631-642. doi:10.1177/03635465231219261
211. Tran P, Pritchard M, O'Donnell J (2013) Outcome of arthroscopic treatment for cam type femoroacetabular impingement in adolescents. ANZ J Surg 83 (5):382-386. doi:10.1111/j.1445-2197.2012.06197.x
212. Ukwuani GC, Waterman BR, Nwachukwu BU, Beck EC, Kunze KN, Harris JD, Nho SJ (2019) Return to Dance and Predictors of Outcome After Hip Arthroscopy for Femoroacetabular Impingement Syndrome. Arthroscopy 35 (4):1101-1108.e1103. doi:10.1016/j.arthro.2018.10.121
213. Vahedi H, Aalirezaie A, Rolo G, Parvizi J (2019) Hip Dysplasia Compromises the Outcome of Femoroacetabular Impingement Surgery. J Arthroplasty 34 (5):852-856. doi:10.1016/j.arth.2019.01.028
214. Vahedi H, Aalirezaie A, Schlitt PK, Parvizi J (2019) Acetabular Retroversion Is a Risk Factor for Less Optimal Outcome After Femoroacetabular Impingement Surgery. J Arthroplasty 34 (7):1342-1346. doi:10.1016/j.arth.2019.02.050
215. Varshneya K, Abrams GD, Sherman SL, Safran MR (2022) Patient-Specific Risk Factors Exist for Hip Fractures After Arthroscopic Femoroacetabular Impingement Surgery, But Not for Dislocation-An Analysis of More Than 25,000 Hip Arthroscopies. Arthrosc Sports Med Rehabil 4 (2):e519-e525. doi:10.1016/j.asmr.2021.11.011
216. Wang WG, Yue DB, Zhang NF, Hong W, Li ZR (2011) Clinical diagnosis and arthroscopic treatment of acetabular labral tears. Orthop Surg 3 (1):28-34. doi:10.1111/j.1757-7861.2010.00121.x
217. Wang AS, Lamba A, Okoroha KR, Levy BA, Krych AJ, Hevesi M (2023) Long-Term Outcomes of Primary Hip Arthroscopy With Labral Repair for Femoroacetabular Impingement: Results at Minimum 9-Year Follow-up. Orthop J Sports Med 11 (10):23259671231204337. doi:10.1177/23259671231204337
218. Weber AE, Nakata H, Mayer EN, Bolia IK, Philippon MJ, Snibbe J, Romano R, Tibone JE, Gamradt SC (2020) Return to Sport After Hip Arthroscopy for Femoroacetabular Impingement Syndrome in NCAA Division I Athletes: Experience at a Single Institution. Orthop J Sports Med 8 (5):2325967120918383. doi:10.1177/2325967120918383
219. White BJ, Patterson J, Scoles AM, Lilo AT, Herzog MM (2020) Hip Arthroscopy in Patients Aged 40 Years and Older: Greater Success With Labral Reconstruction Compared With Labral Repair. Arthroscopy 36 (8):2137-2144. doi:10.1016/j.arthro.2020.04.031
220. White BJ, Spears H, McKaughan Q, Constantinides SM (2023) Treatment of Severe Pincer-Type Femoroacetabular Impingement With

Arthroscopic Significant Acetabular Rim Correction and Circumferential Labral Reconstruction Improves Patient-Reported Outcome Measures. Arthroscopy 39 (1):41-50. doi:10.1016/j.arthro.2022.05.014
221. Winge S, Winge S, Kraemer O, Dippmann C, Hölmich P (2021) Arthroscopic treatment for femoroacetabular impingement syndrome (FAIS) in adolescents-5-year follow-up. J Hip Preserv Surg 8 (3):249-254. doi:10.1093/jhps/hnab051
222. Wirries N, Ezechieli M, Schwarze M, Derksen A, Budde S, Ribas M, Windhagen H, Floerkemeier T (2022) The lateral joint space width is essential for the outcome after arthroscopically assisted mini-open arthrotomy for treatment of a femoroacetabular impingement: an analysis of prognostic factors for the success of this hip-preserving technique. Int Orthop 46 (2):205-214. doi:10.1007/s00264-021-05181-4
223. Yacovelli S, Sutton R, Vahedi H, Sherman M, Parvizi J (2021) High Risk of Conversion to THA After Femoroacetabular Osteoplasty for Femoroacetabular Impingement in Patients Older than 40 Years. Clin Orthop Relat Res 479 (5):1112-1118. doi:10.1097/corr.0000000000001554
224. Yang F, Huang HJ, He ZY, Xu Y, Zhang X, Wang JQ (2022) Extent of Cam Resection Relative to Epiphyseal Line and Its Association With Clinical Outcomes After Arthroscopic Treatment for Femoroacetabular Syndrome. Orthop J Sports Med 10 (9):23259671221125509. doi:10.1177/23259671221125509
225. Yang F, Shi Y, Zhang Z, Zhang X, Huang H, Ju X, Wang J (2023) Arthroscopy Confers Excellent Clinical Outcomes in Femoroacetabular Impingement Syndrome (FAIS) Patients Aged 50 Years and Above. Orthop Surg 15 (4):947-952. doi:10.1111/os.13666
226. Yang F, Shao J, Zheng X, Bi G, Zhang X, Huang H, Wang J (2023) Does Preoperative Activity Level Affect Postoperative Clinical Outcomes Following Hip Arthroscopy in Femoroacetabular Impingement Syndrome (FAIS) Patients? Orthop Surg 15 (7):1756-1762. doi:10.1111/os.13768
227. Yin QF, Wang L, Liang T, Zhao H, Wang XS (2021) Longitudinal Capsulotomy in Hip Arthroscopy: A Safe and Feasible Procedure for Cam-Type Femoracetabular Impingement. Orthop Surg 13 (6):1793-1801. doi:10.1111/os.13041
228. You JS, Flores SE, Friedman JM, Lansdown DA, Zhang AL (2020) The Learning Curve for Hip Arthroscopic Surgery: A Prospective Evaluation With 2-Year Outcomes in Patients With Femoroacetabular Impingement. Orthop J Sports Med 8 (10):2325967120959140. doi:10.1177/2325967120959140
229. Zacharias AJ, Dooley M, Mosiman S, Spiker AM (2024) Depression Scores Decrease After Hip Arthroscopy for Femoroacetabular Impingement Syndrome. Arthrosc Sports Med Rehabil 6 (2):100871. doi:10.1016/j.asmr.2023.100871
230. Zhang S, Dong C, Li Z, Wang Z, Wei M, Tong P, Li C (2021) Endoscopic Iliotibial Band Release During Hip Arthroscopy for Femoroacetabular Impingement Syndrome and External Snapping Hip Had Better Patient-Reported Outcomes: A Retrospective Comparative Study. Arthroscopy 37 (6):1845-1852. doi:10.1016/j.arthro.2021.01.040
231. Zimmerer A, Schneider MM, Nietschke R, Miehlke W, Sobau C (2020) Is Hip Arthroscopy an Adequate Therapy for the Borderline Dysplastic Hip? Correlation Between Radiologic Findings and Clinical Outcomes. Orthop J Sports Med 8 (5):2325967120920851. doi:10.1177/2325967120920851
232. Zimmerer A, Ramoser A, Streit M, Janz V, Sobau C, Wassilew GI, Miehlke W (2021) Osteoarthrosis, Advanced Age, and Female Sex Are Risk Factors for Inferior Outcomes After Hip Arthroscopy and Labral Debridement for Femoroacetabular Impingement Syndrome: Case Series With Minimum 10-Year Follow-Up. Arthroscopy 37 (6):1822-1828.e1821. doi:10.1016/j.arthro.2021.01.024
233. Zimmerer A, Janz V, Sobau C, Wassilew GI, Miehlke W (2021) Defining the Clinically Meaningful Outcomes for Arthroscopic Treatment of Femoroacetabular Impingement Syndrome at Minimum 10-Year Follow-up: The Timing of Surgery Is Crucial. Orthop J Sports Med 9 (2):2325967120985140. doi:10.1177/2325967120985140
